# Microplastics in Internal Tissues of Companion Animals from Urban Environments

**DOI:** 10.3390/ani12151979

**Published:** 2022-08-04

**Authors:** Joana C. Prata, Ana L. Patrício Silva, João P. da Costa, Patrícia Dias-Pereira, Alexandre Carvalho, António José Silva Fernandes, Florinda Mendes da Costa, Armando C. Duarte, Teresa Rocha-Santos

**Affiliations:** 1 Centre for Environmental and Marine Studies (CESAM) & Department of Biology, University of Aveiro, 3810-193 Aveiro, Portugal; 2 Centre for Environmental and Marine Studies (CESAM) & Department of Chemistry, University of Aveiro, 3810-193 Aveiro, Portugal; 3 Department of Pathology and Molecular Immunology, Institute for the Biomedical Sciences Abel Salazar, Porto University (ICBAS-UP), 4050-313 Porto, Portugal; 4 I3N & Physics Department, University of Aveiro, 3810-193 Aveiro, Portugal

**Keywords:** small animals, pets, microplastics in biota, Nile Red

## Abstract

**Simple Summary:**

Microplastics are widespread anthropogenic contaminants, imposing a potential threat to organisms. A preliminary study was conducted to assess microplastics in postmortem samples of internal tissues of companion animals. Suspected microplastics were observed in the internal tissues of cats and dogs. Suspected microplastics were found in 35 out of 49 animals and 80 out of 242 samples. Particles sized 1–10 µm comprised 50.3% of the suspected microplastics. The number of particles found was very low and analytical methods must still be developed to improve the characterization and quantification of smaller-sized factions of microplastics. Moreover, this study suggests that microplastics may be internalized and distributed to the internal tissues of terrestrial vertebrates.

**Abstract:**

Companion animals living in urban areas are exposed to environmental contaminants, which may include microplastics. A preliminary study was conducted by collecting postmortem samples from the internal tissue (lungs, ileum, liver, kidney, and blood clots) of 25 dogs (*Canis familiaris*) and 24 cats (*Felis catus*) living in an urban environment in Porto metropolitan area, Portugal. Suspected microplastics were found in 80 samples from 35 animals (18 cats and 17 dogs), often occurring in more than one tissue of the same animal (71.4%), primarily under small sizes (50.3% as 1–10 µm). Micro-Raman spectroscopy confirmed a fraction of particles as common polymer types (e.g., polyethylene terephthalate). However, the number of particles was very low. This study highlights the possibilities of the internalization and distribution of microplastics in the internal tissues of terrestrial vertebrates.

## 1. Introduction

Microplastics are persistent and ubiquitous anthropogenic contaminants commonly found in urban environments [1]. Plastic particles < 5 mm, classified as microplastics, can be industrially produced to be used in consumer products or anthropogenetic activities (primary microplastics) or result from the weathering and physical degradation of larger plastics (secondary microplastics) [2]. Concerns over microplastics stem from their potential adverse effects on organisms as a result, for instance, of the formation of reactive oxygen species and consequent oxidative stress and inflammation [3] and accumulation in the environment, with potential for disrupting Earth system processes [4]. Respiratory and dietary exposure are thought to be the major exposure routes for mammals, with microplastics potentially causing abrasive lesions and inflammatory responses, releasing adsorbed chemicals, or harboring opportunistic pathogens [5]. Microplastics have been found in the digestive systems of fish [6], mussels [7], birds [8], and marine mammals [9] but also in the internal organs of fish (e.g., liver), suggesting translocation [10]. Indeed, translocation from the lumen to the intra- and intercellular spaces and distribution to other internal tissues may occur for smaller microplastics, for instance, in Peyer’s patches or by paracellular transference [11,12]. However, studies of microplastics on internal tissues are scarce, even for well-studied species such as fishes [13]. Using pyrolysis gas chromatography mass spectrometry after filtering through a >10 µm membrane, a study on wild coastal animals of Norway found microplastics in the internal tissues (e.g., stomach, intestine, liver, muscle) of otters (*Lutra lutra*), birds (*Mergus serrator*, *Uria aalgae*), and fish (*Gadus morhua*, *Limanda limanda*) [14]. Microplastics found in this study were mainly of polyvinyl chloride, polystyrene and polyethylene terephthalate (PET) [14]. Although studies have been conducted on wild animals, little information is available on the presence of microplastics in the internal tissues of domestic animals. Microplastics of PET and polycarbonate (PC) have been found in pet food and the feces of cats and dogs [15]. In this study, PET was found in median concentrations of 61,000 and 30,000 ng g^−1^ in cat and dog feces, respectively. Considering the median concentrations of PET in pet foods (<1500 ng g^−1^), dietary exposure seems to be only a small contributor. Thus, companion animals living in urban environments can experience dietary and environmental exposure to microplastics, which has so far, to the best of the authors’ knowledge, not been addressed. The current study aimed to conduct a preliminary assessment of the presence of microplastics in internal tissues, namely the lungs, small intestine (ileum), kidney, liver, and blood clots of domestic cats and dogs living in an urban environment by collecting postmortem samples and using Nile Red staining (i.e., suspected microplastics) and micro-Raman spectroscopy (i.e., confirmed microplastics). Specifically, the objectives were to: (i) provide insights on the characteristics of internalized suspected microplastics; (ii) understand the distribution of suspected microplastics in the internal tissues of domestic terrestrial mammals; (iii) test the influence of the postmortem findings related to cause of death and age groups on the concentrations in internal tissues; and (iv) identify knowledge gaps that need to be addressed before a larger sampling effort.

## 2. Materials and Methods

### 2.1. Sample Characterization

Cats (*Felis catus*) and dogs (*Canis familiaris*) from the Porto metropolitan area (Portugal) were subjected to necropsy examination in 2019, upon the owner’s requests and consent, as part of veterinary care and services provided by the Instituto de Ciências Biomédicas Abel Salazar (ICBAS), University of Porto. For each pet, a necropsy request form was signed by the veterinary clinician and the pet owner, comprising an informed consent to perform the clinical necropsy (and any necessary histopathological exams), allowing use for teaching and research purposes, and providing a clinical history background (Figure S3, Supplementary Materials). Animals were classified according to age (junior < 1 year, adult 1–7 years, senior ≥ 8 years) and cause of death determined by postmortem findings (respiratory, urinary, digestive system, cancer, or other). Cats varied from 1.6 to 6 kg body weight for adults and seniors, respectively, both being European shorthairs. Dogs varied from a 0.2 kg junior English Bulldog to a 53.6 kg adult Bernese Mountain Dog. More information on sample characterization is provided in Table S1, Supplementary Materials. Sampled organs were chosen to represent exposure routes, namely deep lung and gastrointestinal exposure in the small intestine (namely in the ileum abounding in Peyer Patches where internalization is thought to occur), distribution through the blood, and detoxification and excretion routes through the liver and kidney. Fragments, namely the tip from the caudal area of the lung, ileum (~5 cm of the proximal section, small intestine), liver, kidney (including all layers), and blood clot collected from the ventricular cavity (absent from 2 cats and 1 dog), were collected from 24 cats and 25 dogs in a glass flask using metal instruments (i.e., tweezers and scissors), providing a snapshot of the distribution of particles in the organism under real world conditions.

### 2.2. Sample Preparation

Fragments, except blood clots, were washed with a jet of filtered distilled water inside the laminar flow hood to remove any surface contamination and all samples were weighed inside closed glass flasks to the nearest 0.1 mg (Sartorius, Entris, Germany). A method for preparing biological samples for Nile Red staining, previously developed and tested, was followed [16]: (i) incubation for 24 h at 60 °C (Oasis™ Benchtop IR CO_2_ Incubator, Caron, OH, USA) in 30 mL of 10% KOH (*w*/*v*, ≥85%, Labchem, PA, USA); (ii) at 24 h, the temperature was raised to boiling point and the solution immediately filtered on glass fiber filter membrane (1.2 µm pore, Whatman^®^ GF/C, Little Chalfont, Buckinghamshire, UK) mounted in a vacuum glass filtration system; (iii) 100 mL of boiling filtered distilled water was filtered to remove soaps; (iv) 10 mL of acetone was added to the cup for 10 min; (v) an additional 10 mL of acetone was filtered to remove lipophilic matter; (vi) 0.5–1 mL of 0.01 mg mL^−1^ of Nile Red (microscopy grade, Sigma-Aldrich, St. Louis, MO, USA) was added to the cup for 5 min; (vii) 50 mL of distilled water was filtered; and (viii) stored in glass Petri dishes kept inside cardboard boxes for a week to dry at room temperature (20 °C).

### 2.3. Sample Analysis

Particles were considered suspected microplastics (1–5000 µm) if presenting Nile Red fluorescence, tridimensional shape, and well-defined edges when observed under the 10x microscope objective of the optical microscope (Olympus BX41, Tokyo, Japan) illuminated by an external 470 nm FOCUS LED (SPEX Forensic, Piscataway, NJ, USA) and a digital camera (Canon 1200D, Tokyo, Japan) attached to an orange camera lens filter (Standard ProMaster^®^ Orange Filter, Fairfield, CT, USA) [17,18]. Quantification was performed by photographing particles in the two lanes of each filter membrane (23% of the area, adapted from [16]), which were individually measured in ImageJ, and their numbers used to estimate the total number of particles. A fraction of the fluorescent particles resulting from Nile Red staining in filters were analyzed by micro-Raman spectroscopy using the 100× objective with a numerical aperture of 0.9 of a Horiba (Jobin-Yvon, Bensheim, Germany) HR800 micro Raman instrument (Tokyo, Japan) with a 442 nm line of an external He:Cd laser, and selecting acquisition conditions varying from 10–30 accumulations, 1–2 s, in 2 spectral zones (in a total of 20–120 s), and compared to reference material spectra and to the OpenSpecy library [19]. 

### 2.4. Contamination Control Measures

Strict contamination control measures were conducted, namely by: (i) wearing cotton lab coats and clean gloves; (ii) working in the laminar flow hood in a clean room, maintained by cleaning with ethanol in paper towels followed by cleaning with a duster that attracts and traps dust particles and paper fibers; (iii) filtering all solutions (1.2 µm pore, Whatman^®^ GF/C); (iv) using glass and metal materials; (v) decontaminating glass materials by submerging them in 10% HNO_3_ for at least 30 min and rinsing with distilled water; (vi) additionally cleaning glass Petri dishes with a N_2_ air jet; (vii) burning glass microfiber filter membranes at 450 °C for 3 h; (viii) cleaning the cup of the filtration system between samples by dipping it for 15 s in a solution (first batch: acetone, second batch: 30% HNO_3_) followed by dipping in distilled water; (ix) keeping sample containers closed as much as possible with glass lids, caps, or aluminum foil; and (x) conducting procedural blanks (solutions without samples exposed to sample preparation procedures) for each batch. 

### 2.5. Data Analysis and Statistics

Samples were processed in two batches. Considering expected internalized sizes, especially smaller particles that are more likely to cross biological barriers, suspected microplastics (i.e., lacking chemical characterization) were divided into five particle size categories (]1, 10], ]10, 20], ]20, 50], ]50, 100], ]100, 5000] µm), based on the equivalent diameter (hereby diameter) of a circle with a similar area, as determined by ImageJ. Concentrations were calculated based on the sample wet weight (since only a fragment of the organ, containing all layers, was used) and after subtracting medians of blanks, considering size categories, and batches (Table S2). Particle shape was inferred from circularity as defined by ImageJ, varying from an elongated shape (0.0) to a perfect circle (1.0). Sample weight loss (%), corresponding to the digestion efficiency, was calculated as 100 × W_i_ − W_f_)/W_i_, where W_i_ and W_f_ are the initial and final weight of the sample, respectively [16]. Data were recorded in Excel and statistical analysis was performed in IBM SPSS Statistics 26, namely descriptive statistics and non-parametric analysis of variance. Kruskal–Wallis tests followed by pairwise comparisons with Bonferroni corrections, considering α = 0.05, were used to compare (i) the equivalent diameter, circularity, largest particle dimension, and smallest particle dimension by internal tissue; (ii) equivalent diameter by tissue split by species; (iii) equivalent diameter by species split by tissue; (iv) microplastics concentration by cause of death split by tissue; and (v) microplastics concentrations by age categories split by tissue (Table 1). Additional information and a record of the suspected microplastics are presented in the Supplementary Materials.

## 3. Results and Discussion

The current work examined the presence of suspected microplastics in cats and dogs living in the Porto metropolitan region. Suspected microplastics were found in 35 animals (18 cats and 17 dogs) and 80 samples, meaning that microplastics were not necessarily detected in all 5 tissues of the same animal. Out of 49 samples of each tissue, after blank corrections, suspected microplastics were detected in 22 samples of the kidney, 19 of the lungs, 17 of the ileum, and 14 of the liver (Table S3). Suspected microplastics were also detected in 8 out of 46 samples of blood clots (missing in 3 animals), possibly due to the dynamics of blood flow, clot formation, and particle distribution. As a medium-sized European city, Porto is an urban environment with a high density of anthropogenic pressures (e.g., traffic, high population density) potentially leading to microplastic exposure (e.g., respiratory exposure). Exposure could also have occurred through the ingestion of contaminated food [15] or water [20], cleaning behaviors after particle deposition in the fur, or chewing and ingestion of plastic foreign material [21]. However, the numbers of suspected microplastics in tissues resulted in a median of zero particles (Table 2), and therefore median concentrations of 0 MP g^−1^ (Table S6). One exception is the concentrations found in blood clots for smaller particle sizes (1–20 µm), which should be interpreted by accounting for the low sample weights, the concentrated nature of this sample, and the amount of blood volume present in the animal (e.g., 66 mL kg^−1^ of body weight in cats [22]). No significant differences in the concentration of suspected microplastics were found according to the cause of death, despite the potential of inflammation to increase the permeability and thus the internalization of particles, nor with age groups, which could predispose to accumulation (Table 1).

Most suspected microplastics found were sized between 1 and 10 µm, comprising 69.4% of suspected microplastics in the ileum, 53.5% in the lungs, 44.2% in the kidney, 42.6% in the blood clot, and 26.7% in the liver (Table 2). This translated into a distribution of 50.3% of particles ]1, 10], 33.4% of ]10, 20], 12.6% of ]20, 50], and 3.7% of ]50, 100] µm. Smaller sizes are more abundant in the environment due to the progressive fragmentation of microplastics [23] and have a higher probability of translocation into internal tissue due to the effect of biological barriers [10]. All suspected microplastics presented sizes ranging between 1.3 and 93.9 µm (Table S4), limited by screening conditions (1 µm limit) and biological barriers, considering restricted internalization for particles > 150 µm [24,25]. In the intestine, uptake may occur through paracellular transfer, limited in size by intercellular adhesion mechanisms [11,26], and through Peyer’s patches and/or translocation to the lymphatic system, including for particles ≥ 100 µm [25,27]. Absorption in the dog’s gut of 3–100 µm inert starch microparticles [28] also supports absorption of inert polymeric materials. For instance, administration of 200 g of 5–110 µm polyvinyl chloride microbeads *per os* in dogs resulted in rapid internalization and distribution peaking at 30 µm, with faster elimination of larger sizes [29]. It has been suggested that only particles < 20 µm may be able to penetrate into the organs [25], from local vessels into interstitial tissue, but such differentiation can only be attainable by histopathology. Moreover, the presence of an eco-corona in environmental microplastics may promote internalization (i.e., the presence of surface-adsorbed biomolecules may facilitate translocation through biological barriers), supporting differences between laboratory exposures based on virgin polymers and biota samples [30]. The internalization of larger particles can also occur through piercing wounds, as described in companion animals for millimetric plant material or wood, often resulting in inflammation and infection [31,32,33]. Larger sizes may also be an artifact from particle aggregation.

Significant differences in sizes were found between blood clots (median 10.3 µm) and the lungs (6.0 µm) or ileum (4.5 µm),and between the ileum (4.5 µm) and kidney (8.5 µm) but not the liver (7.6 µm) and generally in cats compared to dogs, with the felines presenting larger sizes possibly due to differences in sample size (i.e., higher number of particles found in cats) and anatomy or susceptibility (Table 1, Table S5). For instance, indoor PM2.5 has been related to respiratory disease in cats but not in dogs [34]. Internal organs can be reached through the systemic circulation, with 71.4% of cases being distributed to more than one tissue of the individual, and considering that suspected microplastics in blood clots presented sizes similar to hematocytes (i.e., 5–15 µm) [35]. Laboratory assays support, as a proof of concept, the distribution of microplastics to the gut, liver, and kidney of mice (*Mus musculus*, 0.1 mg day^−1^ of 5 and 20 µm PS) [36] and lungs, liver, spleen, kidney, and heart of dogs (3–24 µm radiolabeled polystyrene divinylbenzene microspheres, PS-DB) [37] after oral and intravenous administration, respectively. Bidirectional exchange might occur between the blood and tissues, possibly contributing to the smaller sizes found in the latter. Indeed, microspheres in circulation can accumulate in the narrower capillaries, causing mechanical blockage, potentiated by particle aggregation [38], or suffer differential removal by phagocytes or platelet aggregation [39]. Larger particle sizes may be found in the kidney due to accumulation in the preglomerular afferent arterioles, with a diameter of 18 µm in the dog [40]. Similarly, in addition to exposure to only a smaller inhalable size (<10 µm), the lung presents a narrow pulmonary capillary network and slower elimination of smaller microplastics [41], with retention of polystyrene microspheres > 8 µm injected in the pulmonary artery of dogs, being unable to cross the pulmonary capillaries [42]. Excretion may occur: (i) in the liver, captured by hepatocytes (potentially with temporary accumulation) followed by exocytosis in the bile and possible reabsorption in the intestine (enterohepatic circulation) [43]; (ii) by the phagocytes of the reticuloendothelial system followed by elimination by exocytosis in the gut lumen [44]; or (iii) by trapping in the splenic interendothelial cell slits [45]. Elimination may be swifter for larger particles in circulation, which are more easily recognized [46], as exemplified by the faster distribution of PS-DB ≥ 7.4 µm to the liver and spleen than 3.4 µm, following intravenous administration in dogs [41].

**Figure 1:**
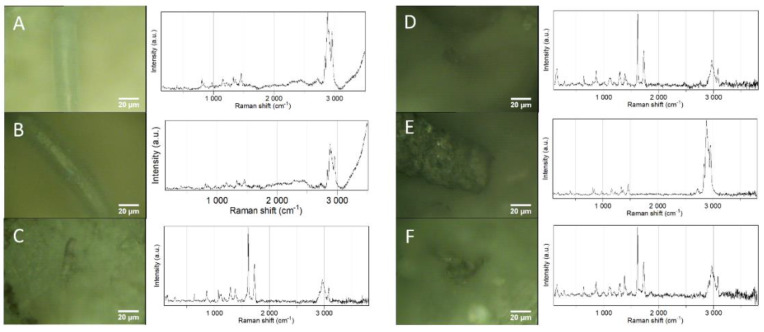
Raman spectra of microplastics found in the filters of samples of internal tissues of different companion animals: (**A**,**B**) polypropylene particle in cat liver; (**C**) polyethylene terephthalate particle in cat ileum; (**D**) polyethylene terephthalate in cat blood clot; (**E**) polypropylene particle in dog lung; (**F**) polyethylene terephthalate particle in dog lung. Edited in Adobe Photoshop CS6 to improve brightness and contrast by 50 and 30, respectively. Original colors can be found in Figure S2, Supplementary Materials.

Median circularity, describing how a shape varies between an elongated (0.000) and a perfect circle (1.000), was 0.873 (0.265–0.988), which did not differ between tissues (H_4_ = 5.733, *p* = 0.218, *n* = 617). Circularity translated into 0.5% fibers (0.0–0.300), 6.3% fragments (0.300–0.600), 58.2% rounded particles (0.600–0.900), and 35.0% spheres (0.900–1.0). Smaller particles tend to present a rounded to spherical shape, justifying the prevalence of these shapes. Micro-Raman spectroscopy allowed chemical characterization of six suspected particles, allowing polymer characterization. As depicted in Figure 1, polypropylene and polyethylene terephthalate were found in the internal tissues of companion animals, reflecting polymer types that are commonly found in households, such as the ones with the highest demand and applications [47], and also including types previously reported in pet food and the feces of cats and dogs (e.g., PET) [15]. Despite the predominance of particles in the size range of 1–10 µm (50.3%) in companion animal samples, and the theoretical resolution limit of 1 µm for micro-Raman spectroscopy [48], Raman spectra were only successfully collected for microplastics > 20 µm due to difficulties in focusing and obtaining a signal from smaller particles, even after proper sample preparation. Raman spectra could not be collected for rounded and spherical shapes as they mainly comprised particles < 20 µm.

While being the first study conducted to determine suspected microplastics in the internal tissues of companion animals, to the best of the authors’ knowledge, limitations include: (i) the restricted number of animals (*n* = 49), dependent on the availability of necropsy requests, variations between the organisms (e.g., weights, ages), voluntary information provided, and the feasibility of processing a large number of samples in a short amount of time, despite previous efforts on method simplification; (ii) being restricted to the Porto metropolitan area while exposure may vary between different urban environments and lifestyles; (iii) lack of availability of histological techniques for screening microplastics in internal tissues to distinguish between their presence in interstitial tissue or blood vessels; (iv) potential for misidentification of small suspected microplastics screened using staining dyes, considering that unexpected remains of biogenic organic matter can be stained (e.g., liposomes), minimized by identification criteria (e.g., tridimensional shape); (v) the presence of darker coloration in filters, which could have hindered suspected microplastic screening, although less pronounced at the microscopic scale; (vi) and the inability of micro-Raman spectroscopy to be applied to all particles and/or to smaller particle sizes (i.e., <20 µm). Stemming from this study, three major knowledge gaps on methodologies must be addressed to provide a better understanding of microplastics in animal tissues. First, sample processing and identification methods for microplastics still require more development, especially for smaller-sized fractions (<100 µm). Second, biological matrices are complex and may require further development in sampling and sample preparation (e.g., biogenic organic matter removal), depending on the organism and sample characteristics. Third, the development of histological techniques that can preserve and detect microplastics is required for a clear understanding of the exact location in tissues.

## 4. Conclusions

The preliminary results of 24 cats and 25 dogs from the Porto metropolitan area reveal the presence of none to low concentrations of microplastics (median 0 MP g^−1^) characterized by the most common polymer types (e.g., PP, PET) in some samples of internal tissues (e.g., 22 out of 49 of kidney samples), generally under small sizes that are capable of internalization (e.g., 50.3% under 1–10 µm). The distribution and characteristics of the suspected microplastics in tissues were consistent with the expected physiological mechanisms (e.g., particles in circulation comparable to blood cell sizes), often present in more than one tissue of the same individual (71.4%). While limited by sample size, no evidence was found to support accumulation in older animals nor relation to the cause of death. This study also helps to understand key knowledge gaps when working with animal samples, namely the need for developing sample processing and identification methods (particularly for particles < 100 µm), further improving sampling and sample preparation for biological matrices, and developing histological techniques adapted for microplastics sampling.

## Figures and Tables

**Table 1:** Statistical analysis results of the non-parametric Kruskal–Wallis test followed by pairwise comparison with Bonferroni correction.

Analysis	Results
Independent: Internal Tissue Dependent: Equivalent Diameter	H(4) = 41.411, *p* < 0.001, *n* = 617 Ileum—kidney: *p* = 0.001 Ileum—liver: *p* = 0.039 Blood clot—ileum: *p* < 0.001 Blood clot—lungs: *p* < 0.001 Blood clot—kidney: *p* = 0.058
Independent: Internal Tissue Dependent: Largest dimension	H(4) = 36.432 *p* < 0.001, *n* = 617 Ileum—kidney: *p* = 0.001 Blood clot—ileum: *p* < 0.001 Blood clot—lungs: *p* < 0.001
Independent: Internal Tissue Dependent: Smallest dimension	H(4) = 42.228, *p* < 0.001, *n* = 617 Ileum—kidney: *p* = 0.001 Ileum—liver: *p* = 0.024 Blood clot—ileum: *p* < 0.001 Blood clot—lungs: *p* < 0.001 Blood clot—kidney: *p* = 0.034
Independent: Internal Tissue Dependent: Circularity	H(4) = 6.375 *p* = 0.173, *n* = 617
Independent: Species Dependent: Equivalent Diameter Split: Tissue	Liver: H(1) = 1.494, *p* = 0.222, *n* = 63 Lungs: H(1) = 5.383, *p* = 0.020, *n* = 118 Kidney: H(1) = 4.458, *p* = 0.035, *n* = 152 Ileum: H(1) = 10.475, *p* = 0.001, *n* = 126 Blood clot: H(1) = 4.081, *p* = 0.043, *n* = 88
Independent: Tissue Dependent: Equivalent Diameter Split: Species	Cat: H(4) = 59.177, *p* < 0.001, *n* = 388 Blood clot—lungs: *p* = 0.009 (10.5 vs. 6.8 µm) Ileum—lungs: *p* = 0.001 (3.9 vs. 6.8 µm) Ileum—liver: *p* = 0.001 (3.9 vs. 9.0 µm) Ileum—kidney: *p* < 0.001 (3.9 vs. 9.6 µm) Ileum—blood clot: *p* < 0.001 (3.9 vs. 10.5 µm)
	Dog: H(4) = 4.948, *p* = 0.293, *n* = 229
Independent: Cause of deathDependent: Microplastic concentrationsSplit: Tissue	Liver: H(4) = 9.203, *p* = 0.056, *n* = 49 Lungs: H(4) = 2.232, *p* = 0.693, *n* = 49 Ileum: H(4) = 1.389, *p* = 0.846, *n* = 49 Kidney: H(4) = 3.721, *p* = 0.445, *n* = 49 Blood clot: H(4) = 6.846, *p* = 0.143, *n* = 46
Independent: Age categories Dependent: Microplastic concentrations Split: Tissue	Liver: H(3) = 4.975, *p* = 0.174, *n* = 49 Lungs: H(3) = 0.996, *p* = 0.802, *n* = 49 Ileum: H(3) = 3. 815, *p* = 0.282, *n* = 49 Kidney: H(3) = 2.087, *p* = 0.555, *n* = 49 Blood clot: H(3) = 0.923, *p* = 0.820, *n* = 46

**Table 2:** Median, Min-Max, and Sum of the number of suspected microplastics found in 23% of the sample filter membrane after blank corrections by size categories, and number of individual samples containing suspected microplastics (*n*) per internal tissues of companion animals.

		Suspected Microplastics in 23% of Sample Filter				
Sizes (µm)		Lungs	Blood Clot	Kidney	Ileum	Liver
]1, 10]	Median	0.0	0.0	0.0	0.0	0.0
	Min–Max	(0.0–21.0)	(0.0–23.0)	(0.0–15.0)	(0.0–34.0)	(0.0–3.0)
	Sum	54	26	38	50	8
]10, 20]	Median	0.0	0.0	0.0	0.0	0.0
	Min–Max	(0.0–11.0)	(0.0–16.0)	(0.0–6.0)	(0.0–4.0)	(0.0–4.0)
	Sum	34	23	35	9	16
]20, 50]	Median	0.0	0.0	0.0	0.0	0.0
	Min–Max	(0.0–3.0)	(0.0–5.0)	(0.0–2.0)	(0.0–2.0)	(0.0–2.0)
	Sum	11	10	9	8	6
]50, 100]	Median	0.0	0.0	0.0	0.0	0.0
	Min–Max	(0.0–2.0)	(0.0–1.0)	(0.0–1.0)	(0.0–2.0)	(0.0–0.0)
	Sum	2	2	4	5	0
*n*		19	8	22	17	14

## Data Availability

Data available by consulting the Supplementary Materials.

## Supplementary Materials

The following supporting information can be downloaded at: https://www.mdpi.com/article/10.3390/ani12151979/s1, including additional presentation of Figures S1–S3 and Tables S1–S9 and data availability (library of Figures S4–S150).
