# Supplementary material for: Microplastics in Internal Tissues of Companion Animals from Urban Environments
Source: Animals (Basel). 2022 Aug 4;12(15):1979. doi: 10.3390/ani12151979 (PMC9367336; doi:10.3390/ani12151979)
Supplement: Supplementary file 1 [file animals-12-01979-s001.zip › animals-1818800-supplementary.pdf]

Microplastics in internal tissues of companion animals from urban environments

Supplementary Material

Table S1. Characteristics of samples companion animals.

Characteristic	Cats	Dogs
Sex	15 Females<br>9 Males	9 Females<br>16 Males
Age	6 adults (1 – 7 years)<br>17 seniors (≥8 years)<br>1 undetermined	1 junior (<1 year)<br>7 adults (1 – 7 years)<br>15 seniors (≥8 years)<br>2 undetermined
Breeds	European shorthairs (22), Persian (1), Persian crossbreed (1).	Mix-breed (12), Cocker Spaniels (3), Basset Hounds (2), Labradors (2), Shar-pei (1), English Bulldog (1), French Bulldog (1), Bernese Mountain Dog (1), Belgian Shepherd (1), Pitbull crossbreed (1).
Cause of death	Respiratory system (4), urinary system (7), digestive system (1), cancer (6), others (6)	Respiratory system (4), urinary system (5), digestive system (2), cancer (4), others (10)
Stray animals	2	1

Table S2. Number of suspected microplastics found in 23% of the filter of the procedural blanks.

Batch	Blank	]1, 10]	]10, 20]	]20, 50]	]50, 100]	]100, 5000]
1	1	12	3	0	0	0
	2	6	4	1	0	0
	Median	*9*	*4*	*1*	*0*	*0*
2	1	1	1	0	0	0
	2	0	0	0	0	0
	3	0	0	0	0	0
	4	3	0	0	0	0
	Median	*1*	*0*	*0*	*0*	*0*

Table S3. Samples where suspected microplastics were detected, after blank correction, for each tissue and species, compared to the total of samples.

Species	Liver	Lungs	Ileum	Kidney	Blood clot
Cat	10/24	11/24	7/24	11/24	7/22
Dog	4/25	8/25	10/25	11/25	1/24
Total	14/49	19/49	17/49	22/49	8/46

Table S4. Median particle dimensions (µm) as largest dimension (Feret), smallest dimension (MinFeret), and equivalent diameter calculated from the particle's area.

	Dimension (µm)		
	Largest dimension	Smallest dimension	Equivalent diameter

Lungs	8.1	5.5	6.0
Blood clot	12.1	8.9	10.3
Kidney	10.3	7.7	8.5
Ileum	6.4	3.6	4.5
Liver	9.8	6.7	7.6

Table S5. Median equivalent diameter (µm) for each tissue by species. Median equivalent diameter (µm), median sample wet weight (g), and weight loss (%) corresponding to the efficiency of the digestion procedure for both species.

	Cat		Dog		Total		
Tissue	*n*	Diameter (µm)	*n*	Diameter (µm)	Diameter (µm)	Weight (g)	Weight loss (%)
Lungs	24	6.8	25	5.3	6.0	3.1	99.7
Blood clot	22	10.5	24	6.6	10.3	2.5	97.3
Kidney	24	9.6	25	6.8	8.5	5.1	99.2
Ileum	24	3.9	25	6.7	4.5	2.7	98.1
Liver	24	9.6	25	6.4	7.6	3.9	99.5

Table S6. Median (min – max) of the concentration of suspected microplastics (MP g^{-1}) found in 23% of the sample filter membrane after blank corrections by size categories, and number of individual samples containing suspected microplastics (*n*) per internal tissues of companion animals.

	Median (min-max) of suspected microplastics in 23% of sample filter				
Sizes (µm)	Lungs	Blood clot	Kidney	Ileum	Liver
[1,10]	0.0	19.5	0.0	0.0	0.0
	(0.0 – 20.5)	(0.0 – 282.1)	(0.0 – 35.8)	(0.0 – 17.8)	(0.0 – 4.1)
]10,20]	0.0	22.9	0.0	0.0	0.0
	(0.0 – 13.5)	(0.0 – 196.2)	(0.0 – 14.3)	(0.0 – 35.5)	(0.0 – 4.1)
]20,50]	0.0	0.0	0.0	0.0	0.0
	(0.0 – 8.6)	(0.0 – 168.13)	(0.0 – 4.3)	(0.0 – 10.9)	(0.0 – 1.6)
]50,100]	0.0	0.0	0.0	0.0	0.0
	(0.0 – 4.0)	(0.0 – 52.19)	(0.0 – 4.8)	(0.0 – 8.9)	(0.0 – 0.0)

n	19	8	22	17	14

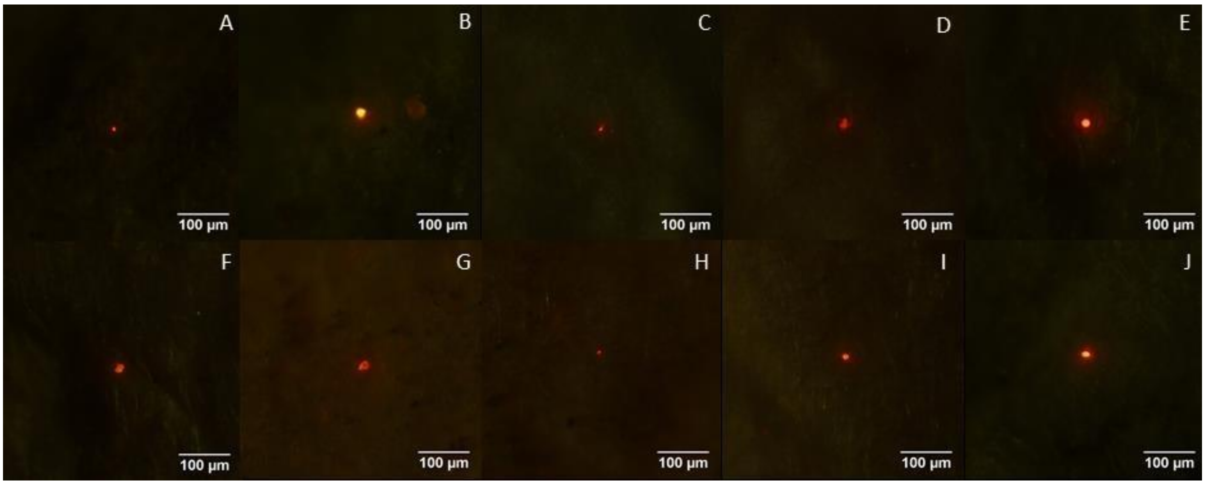

Figure S1. Suspected microplastics in tissues of companion animals (cats and dogs). Examples of microplastics stained with Nile Red and observed under the microscope found in fish: lungs (A, B), blood clots (C, D), kidney (E, F), ileum (G, H), and liver (I, J).

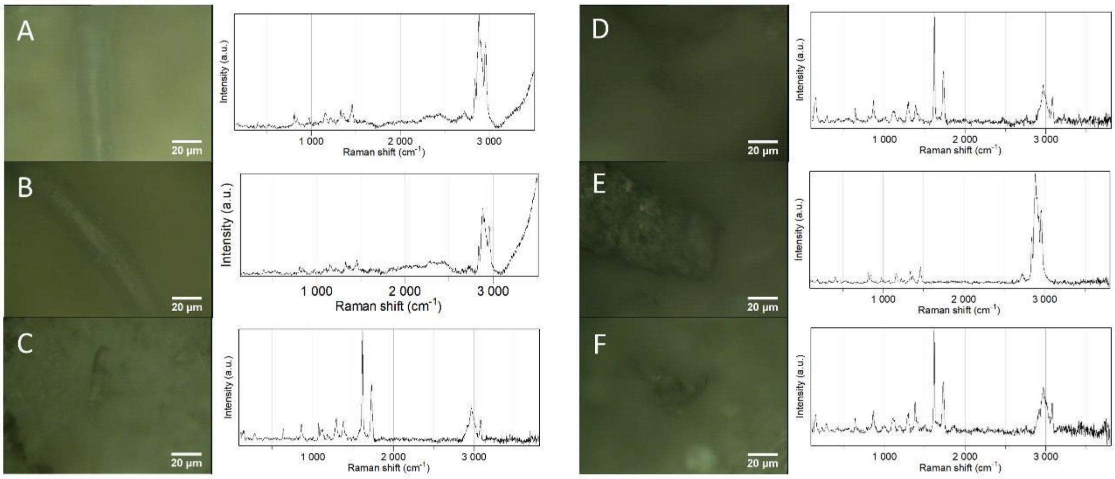

Figure S2. Raman spectra of microplastics found in filters of samples of internal tissues of different companion animal: A and B) polypropylene particle in cat liver; C) polyethylene terephthalate particle in cat ileum; D) polyethylene terephthalate in cat blood clot; E)

polypropylene particle in dog lung; F) polyethylene terephthalate particle in dog lung. Original colors.

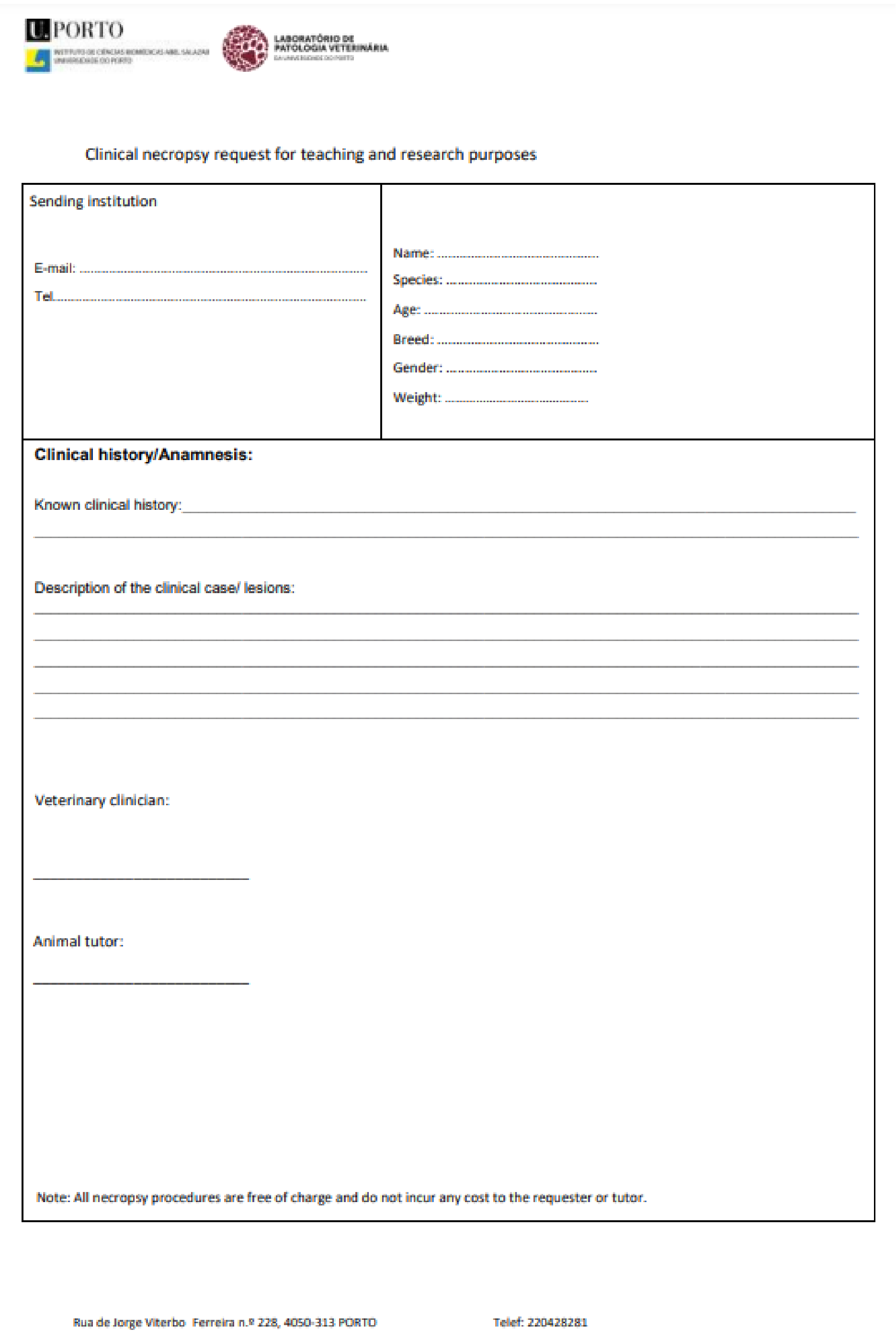

U.PORTO

LABORATÓRIO DE PATOLOGIA VETERINÁRIA

Clinical necropsy request for teaching and research purposes

Sending institution

E-mail: ....

Tel...

Name: ....

Species: ....

Age: ....

Breed: ....

Gender: ....

Weight: ....

Clinical history/Anamnesis:

Known clinical history:____

Description of the clinical case/ lesions:

Veterinary clinician:

____

Animal tutor:

____

Note: All necropsy procedures are free of charge and do not incur any cost to the requester or tutor.

Rua de Jorge Viterbo Ferreira n.º 228, 4050-313 PORTO Telef: 220428281

Figure S3. Blank copy of the necropsy request form, including informed consent sections, signed by both the pet owner and veterinary clinician.

Table S7. Sample coding.

Code	Tissue	Species	Code	Tissue	Species	Code	Tissue	Species	Code	Tissue	Species	Code	Tissue	Species
A001	Liver	Cat	A051	Liver	Cat	A101	Liver	Dog	A151	Ileum	Cat	A201	Kidney	Cat
A002	Lungs	Cat	A052	Lungs	Cat	A102	Lungs	Dog	A152	Blood	Cat	A202	Ileum	Cat
A003	Ileum	Cat	A053	Kidney	Cat	A103	Kidney	Dog	A153	Liver	Cat	A203	Blood	Cat
A004	Kidney	Cat	A054	Ileum	Cat	A104	Ileum	Dog	A154	Lungs	Cat	A204	Liver	Cat
A005	Blood	Cat	A055	Blood	Cat	A105	Blood	Dog	A155	Kidney	Cat	A205	Lungs	Cat
A006	Liver	Cat	A056	Liver	Dog	A106	Liver	Dog	A156	Ileum	Cat	A206	Kidney	Cat
A007	Lungs	Cat	A057	Lungs	Dog	A107	Lungs	Dog	A157	Blood	Cat	A207	Ileum	Cat
A008	Ileum	Cat	A058	Kidney	Dog	A108	Kidney	Dog	A158	Liver	Dog	A208	Blood	Cat
A009	Kidney	Cat	A059	Ileum	Dog	A109	Ileum	Dog	A159	Lungs	Dog	A209	Liver	Dog
A010	Blood	Cat	A060	Blood	Dog	A110	Blood	Dog	A160	Kidney	Dog	A210	Lungs	Dog
A011	Liver	Cat	A061	Liver	Dog	A111	Liver	Cat	A161	Ileum	Dog	A211	Kidney	Dog
A012	Lungs	Cat	A062	Lungs	Dog	A112	Lungs	Cat	A162	Blood	Dog	A212	Ileum	Dog
A013	Kidney	Cat	A063	Kidney	Dog	A113	Kidney	Cat	A163	Liver	Dog	A213	Blood	Dog
A014	Ileum	Cat	A064	Ileum	Dog	A114	Ileum	Cat	A164	Lungs	Dog	A214	Liver	Cat
A015	Blood	Cat	A065	Blood	Dog	A115	Blood	Cat	A165	Kidney	Dog	A215	Lungs	Cat
A016	Liver	Cat	A066	Liver	Dog	A116	Liver	Dog	A166	Ileum	Dog	A216	Kidney	Cat
A017	Lungs	Cat	A067	Lungs	Dog	A117	Lungs	Dog	A167	Blood	Dog	A217	Ileum	Cat
A018	Kidney	Cat	A068	Kidney	Dog	A118	Kidney	Dog	A168	Liver	Cat	A218	Liver	Dog
A019	Ileum	Cat	A069	Ileum	Dog	A119	Ileum	Dog	A169	Lungs	Cat	A219	Lungs	Dog
A020	Blood	Cat	A070	Blood	Dog	A120	Blood	Dog	A170	Kidney	Cat	A220	Kidney	Dog
A021	Liver	Dog	A071	Liver	Cat	A121	Liver	Dog	A171	Ileum	Cat	A221	Ileum	Dog
A022	Lungs	Dog	A072	Lungs	Cat	A122	Lungs	Dog	A172	Blood	Cat	A222	Blood	Dog
A023	Kidney	Dog	A073	Kidney	Cat	A123	Kidney	Dog	A173	Liver	Dog	A223	Liver	Cat
A024	Ileum	Dog	A074	Ileum	Cat	A124	Ileum	Dog	A174	Lungs	Dog	A224	Lungs	Cat
A025	Blood	Dog	A075	Blood	Cat	A125	Liver	Cat	A175	Kidney	Dog	A225	Kidney	Cat
A026	Liver	Dog	A076	Liver	Cat	A126	Lungs	Cat	A176	Ileum	Dog	A226	Ileum	Cat
A027	Lungs	Dog	A077	Lungs	Cat	A127	Kidney	Cat	A177	Blood	Dog	A227	Blood	Cat
A028	Kidney	Dog	A078	Kidney	Cat	A128	Ileum	Cat	A178	Liver	Cat	A228	Liver	Dog
A029	Ileum	Dog	A079	Ileum	Cat	-			A179	Lungs	Cat	A229	Lungs	Dog
A030	Blood	Dog	A080	Blood	Cat	-			A180	Kidney	Cat	A230	Kidney	Dog
A031	Liver	Cat	A081	Liver	Dog	-			A181	Ileum	Cat	A231	Ileum	Dog
A032	Lungs	Cat	A082	Lungs	Dog	-			A182	Blood	Cat	A232	Blood	Dog
A033	Kidney	Cat	A083	Kidney	Dog	A133	Liver	Cat	-			A233	Liver	Dog
A034	Ileum	Cat	A084	Ileum	Dog	A134	Lungs	Cat	A184	Liver	Dog	A234	Lungs	Dog
A035	Blood	Cat	A085	Blood	Dog	A135	Kidney	Cat	A185	Lungs	Dog	A235	Kidney	Dog
A036	Liver	Cat	A086	Liver	Cat	A136	Ileum	Cat	A186	Kidney	Dog	A236	Ileum	Dog
A037	Lungs	Cat	A087	Lungs	Cat	A137	Blood	Cat	A187	Ileum	Dog	A237	Blood	Dog
A038	Kidney	Cat	A088	Kidney	Cat	A138	Liver	Dog	A188	Blood	Dog	A238	Liver	Dog
A039	Ileum	Cat	A089	Ileum	Cat	A139	Lungs	Dog	A189	Liver	Dog	A239	Lungs	Dog
A040	Blood	Cat	A090	Blood	Cat	A140	Kidney	Dog	A190	Lungs	Dog	A240	Kidney	Dog

A041	Liver	Cat	A091	Liver	Cat	A141	Ileum	Dog	A191	Kidney	Dog	A241	Ileum	Dog
A042	Lungs	Cat	A092	Lungs	Cat	A142	Blood	Dog	A192	Ileum	Dog	A242	Blood	Dog
A043	Kidney	Cat	A093	Kidney	Cat	A143	Liver	Dog	A193	Blood	Dog	A243	Liver	Dog
A044	Ileum	Cat	A094	Ileum	Cat	A144	Lungs	Dog	A194	Liver	Dog	A244	Lungs	Dog
A045	Blood	Cat	A095	Blood	Cat	A145	Kidney	Dog	A195	Lungs	Dog	A245	Kidney	Dog
A046	Liver	Cat	A096	Liver	Dog	A146	Ileum	Dog	A196	Kidney	Dog	A246	Ileum	Dog
A047	Lungs	Cat	A097	Lungs	Dog	A147	Blood	Dog	A197	Ileum	Dog	A247	Blood	Dog
A048	Kidney	Cat	A098	Kidney	Dog	A148	Liver	Cat	A198	Blood	Dog			
A049	Ileum	Cat	A099	Ileum	Dog	A149	Lungs	Cat	A199	Liver	Cat			
A050	Blood	Cat	A100	Blood	Dog	A150	Kidney	Cat	A200	Lungs	Cat			

Table S8. Tissue weight (g) per sample code.

Code	Tissue (g)	Code	Tissue (g)	Code	Tissue (g)	Code	Tissue (g)	Code	Tissue (g)
A001	5.30	A051	3.13	A101	7.66	A151	0.49	A201	1.95
A002	2.89	A052	2.54	A102	6.74	A152	0.35	A202	1.75
A003	4.58	A053	6.54	A103	3.54	A153	3.32	A203	0.13
A004	5.11	A054	3.05	A104	4.62	A154	3.23	A204	1.94
A005	1.44	A055	0.03	A105	7.28	A155	2.03	A205	2.15
A006	4.49	A056	12.05	A106	2.31	A156	1.71	A206	12.95
A007	4.35	A057	7.50	A107	3.72	A157	1.03	A207	1.94
A008	2.42	A058	7.82	A108	19.76	A158	13.19	A208	0.33
A009	5.75	A059	13.57	A109	3.24	A159	3.54	A209	2.74
A010	2.02	A060	17.37	A110	5.06	A160	9.25	A210	2.10
A011	1.71	A061	10.72	A111	3.90	A161	2.85	A211	3.82
A012	1.89	A062	4.59	A112	2.42	A162	5.95	A212	1.45
A013	9.28	A063	5.99	A113	5.71	A163	4.23	A213	5.93
A014	2.38	A064	4.96	A114	7.32	A164	3.14	A214	3.80
A015	1.47	A065	10.57	A115	2.73	A165	5.96	A215	1.01
A016	5.28	A066	4.05	A116	3.43	A166	7.56	A216	2.37
A017	4.79	A067	4.65	A117	4.51	A167	16.67	A217	0.80
A018	6.64	A068	6.77	A118	9.96	A168	1.71	A218	1.19
A019	2.82	A069	1.95	A119	5.61	A169	5.89	A219	0.58
A020	0.31	A070	17.29	A120	15.54	A170	5.82	A220	0.91
A021	1.92	A071	3.98	A121	10.09	A171	3.13	A221	0.06
A022	11.24	A072	1.16	A122	9.07	A172	1.08	A222	0.31
A023	5.46	A073	3.69	A123	9.80	A173	9.25	A223	2.15
A024	3.27	A074	0.74	A124	2.21	A174	4.97	A224	1.91
A025	8.35	A075	1.33	A125	3.16	A175	8.07	A225	3.47
A026	8.78	A076	5.22	A126	1.95	A176	3.84	A226	1.52
A027	5.42	A077	4.37	A127	1.94	A177	3.69	A227	0.06
A028	9.18	A078	5.97	A128	2.51	A178	6.77	A228	2.92
A029	4.08	A079	4.07	-	-	A179	1.27	A229	2.18
A030	17.29	A080	1.47	-	-	A180	2.70	A230	4.81
A031	8.96	A081	4.62	-	-	A181	5.21	A231	4.08
A032	3.97	A082	4.51	-	-	A182	1.61	A232	2.87
A033	5.45	A083	6.86	A133	3.85	-	-	A233	2.42

A034	1.49	A084	2.71	A134	2.20	A184	6.76	A234	2.12
A035	0.32	A085	13.90	A135	3.04	A185	3.67	A235	4.92
A036	3.87	A086	14.30	A136	1.00	A186	2.98	A236	1.71
A037	3.12	A087	4.08	A137	2.66	A187	3.76	A237	3.88
A038	1.91	A088	4.16	A138	3.60	A188	3.33	A238	2.50
A039	1.13	A089	12.57	A139	2.10	A189	5.62	A239	2.24
A040	0.20	A090	0.28	A140	2.01	A190	5.16	A240	4.79
A041	6.69	A091	2.24	A141	1.36	A191	3.10	A241	2.39
A042	2.56	A092	1.73	A142	8.07	A192	1.26	A242	2.83
A043	6.93	A093	3.54	A143	6.39	A193	2.44	A243	3.25
A044	2.63	A094	0.78	A144	5.16	A194	3.91	A244	2.46
A045	0.09	A095	0.08	A145	1.82	A195	4.06	A245	7.31
A046	2.37	A096	8.41	A146	3.77	A196	2.88	A246	2.53
A047	2.43	A097	3.22	A147	9.01	A197	5.41	A247	5.00
A048	2.07	A098	7.34	A148	3.03	A198	2.16		
A049	10.89	A099	13.11	A149	1.55	A199	3.66		
A050	0.02	A100	4.59	A150	1.61	A200	2.09		

Table S9. Number of suspected microplastics per sample of animal tissues in 23% of the filter, after corresponding batch blank subtraction, and estimated full filter number (before normalization by sample wet weight).

Sample		Counted in 23%				After blank subtraction				Total after blank subtraction	
**Batch**	**Code**	**]1,10]**	**[10, 20]**	**[20, 50]**	**[50, 100]**	**[1, 10]**	**[10, 20]**	**[20, 50]**	**[50, 100]**	**Total in 23%**	**Total in 100%**
**1**	A001	1	1	3	0	0	0	2	0	2	9
**1**	A002	1	0	0	0	0	0	0	0	0	0
**1**	A003	1	2	0	0	0	0	0	0	0	0
**1**	A004	5	1	2	0	0	0	1	0	1	4
**1**	A005	0	2	0	0	0	0	0	0	0	0
**1**	A006	0	0	0	0	0	0	0	0	0	0
**1**	A007	0	0	1	0	0	0	0	0	0	0
**1**	A008	7	0	1	0	0	0	0	0	0	0
**1**	A009	1	0	0	0	0	0	0	0	0	0
**1**	A010	1	0	0	0	0	0	0	0	0	0
**1**	A011	1	0	0	0	0	0	0	0	0	0
**1**	A012	7	0	0	0	0	0	0	0	0	0
**1**	A013	0	0	0	0	0	0	0	0	0	0
**1**	A014	0	0	0	0	0	0	0	0	0	0
**1**	A015	0	0	1	0	0	0	0	0	0	0
**1**	A016	0	0	0	0	0	0	0	0	0	0
**1**	A017	14	3	2	0	4	0	1	0	5	22
**1**	A018	1	1	0	0	0	0	0	0	0	0
**1**	A019	16	1	0	0	6	0	0	0	6	26
**1**	A020	1	0	0	1	0	0	0	1	1	4
**1**	A021	1	0	1	0	0	0	0	0	0	0
**1**	A022	5	0	0	0	0	0	0	0	0	0
**1**	A023	20	0	0	0	10	0	0	0	10	43

**1**	A024	4	0	0	0	0	0	0	0	0	0
**1**	A025	0	0	0	0	0	0	0	0	0	0
**1**	A026	6	0	0	0	0	0	0	0	0	0
**1**	A027	8	1	0	0	0	0	0	0	0	0
**1**	A028	1	0	0	0	0	0	0	0	0	0
**1**	A029	1	0	0	0	0	0	0	0	0	0
**1**	A030	0	0	0	0	0	0	0	0	0	0
**1**	A031	0	0	0	0	0	0	0	0	0	0
**1**	A032	1	1	0	0	0	0	0	0	0	0
**1**	A033	1	0	0	0	0	0	0	0	0	0
**1**	A034	0	0	0	0	0	0	0	0	0	0
**1**	A035	0	0	0	0	0	0	0	0	0	0
**1**	A036	0	0	0	0	0	0	0	0	0	0
**1**	A037	1	0	1	0	0	0	0	0	0	0
**1**	A038	0	3	0	0	0	0	0	0	0	0
**1**	A039	0	0	0	0	0	0	0	0	0	0
**1**	A040	1	1	0	0	0	0	0	0	0	0
**1**	A041	3	0	0	0	0	0	0	0	0	0
**1**	A042	1	0	0	0	0	0	0	0	0	0
**1**	A043	0	0	0	0	0	0	0	0	0	0
**1**	A044	2	0	0	0	0	0	0	0	0	0
**1**	A045	0	0	0	0	0	0	0	0	0	0
**1**	A046	3	0	0	0	0	0	0	0	0	0
**1**	A047	2	0	0	0	0	0	0	0	0	0
**1**	A048	13	0	0	0	3	0	0	0	3	13
**1**	A049	44	0	0	0	34	0	0	0	34	148
**1**	A050	0	0	0	0	0	0	0	0	0	0
**1**	A051	1	0	0	0	0	0	0	0	0	0
**1**	A052	4	0	2	0	0	0	1	0	1	4
**1**	A053	0	0	0	0	0	0	0	0	0	0
**1**	A054	0	0	0	0	0	0	0	0	0	0
**1**	A055	0	1	0	0	0	0	0	0	0	0
**1**	A056	2	0	0	0	0	0	0	0	0	0
**1**	A057	0	0	0	0	0	0	0	0	0	0
**1**	A058	2	5	2	0	0	1	1	0	2	9
**1**	A059	0	2	1	1	0	0	0	1	1	4
**1**	A060	0	0	0	0	0	0	0	0	0	0
**1**	A061	0	0	0	0	0	0	0	0	0	0
**1**	A062	0	0	0	0	0	0	0	0	0	0
**1**	A063	0	0	0	0	0	0	0	0	0	0
**1**	A064	0	0	0	0	0	0	0	0	0	0
**1**	A065	0	0	0	0	0	0	0	0	0	0
**1**	A066	0	0	0	0	0	0	0	0	0	0
**1**	A067	0	0	0	0	0	0	0	0	0	0
**1**	A068	0	0	0	0	0	0	0	0	0	0
**1**	A069	0	0	0	1	0	0	0	1	1	4
**1**	A070	0	0	0	0	0	0	0	0	0	0
**1**	A071	1	0	0	0	0	0	0	0	0	0

**1**	A072	2	1	2	0	0	0	1	0	1	4
**1**	A073	1	2	1	0	0	0	0	0	0	0
**1**	A074	1	1	0	0	0	0	0	0	0	0
**1**	A075	0	0	0	0	0	0	0	0	0	0
**1**	A076	0	0	0	0	0	0	0	0	0	0
**1**	A077	2	0	0	0	0	0	0	0	0	0
**1**	A078	0	0	0	0	0	0	0	0	0	0
**1**	A079	1	0	0	2	0	0	0	2	2	9
**1**	A080	0	0	0	0	0	0	0	0	0	0
**1**	A081	0	0	0	0	0	0	0	0	0	0
**1**	A082	7	0	0	0	0	0	0	0	0	0
**1**	A083	0	0	0	0	0	0	0	0	0	0
**1**	A084	1	1	0	0	0	0	0	0	0	0
**1**	A085	0	0	0	0	0	0	0	0	0	0
**1**	A086	1	0	0	0	0	0	0	0	0	0
**1**	A087	1	0	0	0	0	0	0	0	0	0
**1**	A088	0	0	1	0	0	0	0	0	0	0
**1**	A089	1	0	0	0	0	0	0	0	0	0
**1**	A090	0	0	0	0	0	0	0	0	0	0
**1**	A091	0	0	0	0	0	0	0	0	0	0
**1**	A092	5	1	0	0	0	0	0	0	0	0
**1**	A093	4	0	0	0	0	0	0	0	0	0
**1**	A094	1	0	0	0	0	0	0	0	0	0
**1**	A095	4	3	2	1	0	0	1	1	2	9
**1**	A096	0	0	0	0	0	0	0	0	0	0
**1**	A097	0	0	0	0	0	0	0	0	0	0
**1**	A098	0	0	0	0	0	0	0	0	0	0
**1**	A099	0	0	0	0	0	0	0	0	0	0
**1**	A100	0	0	0	0	0	0	0	0	0	0
**1**	A101	0	0	0	0	0	0	0	0	0	0
**1**	A102	0	0	0	0	0	0	0	0	0	0
**1**	A103	0	0	0	0	0	0	0	0	0	0
**1**	A104	0	0	0	0	0	0	0	0	0	0
**1**	A105	0	0	0	0	0	0	0	0	0	0
**1**	A106	0	0	0	0	0	0	0	0	0	0
**1**	A107	0	0	0	0	0	0	0	0	0	0
**1**	A108	1	0	0	0	0	0	0	0	0	0
**1**	A109	0	0	0	0	0	0	0	0	0	0
**1**	A110	0	0	0	0	0	0	0	0	0	0
**2**	A111	1	1	0	0	0	1	0	0	1	4
**2**	A112	3	0	0	0	2	0	0	0	2	9
**2**	A113	0	0	0	1	0	0	0	1	1	4
**2**	A114	0	0	0	0	0	0	0	0	0	0
**2**	A115	2	1	0	0	1	1	0	0	2	9
**2**	A116	0	0	0	0	0	0	0	0	0	0
**2**	A117	0	0	0	0	0	0	0	0	0	0
**2**	A118	0	0	0	0	0	0	0	0	0	0
**2**	A119	0	0	0	0	0	0	0	0	0	0

**2**	A120	0	0	0	0	0	0	0	0	0	0
**2**	A121	0	0	0	0	0	0	0	0	0	0
**2**	A122	3	2	0	0	2	2	0	0	4	17
**2**	A123	0	0	0	0	0	0	0	0	0	0
**2**	A124	0	0	1	0	0	0	1	0	1	4
**2**	A125	4	1	0	0	3	1	0	0	4	17
**2**	A126	1	0	0	0	0	0	0	0	0	0
**2**	A127	2	1	0	0	1	1	0	0	2	9
**2**	A128	0	0	0	0	0	0	0	0	0	0
**2**	A129	-	-	-	-	-	-	-	-	-	-
**2**	A130	-	-	-	-	-	-	-	-	-	-
**2**	A131	-	-	-	-	-	-	-	-	-	-
**2**	A132	-	-	-	-	-	-	-	-	-	-
**2**	A133	0	0	0	0	0	0	0	0	0	0
**2**	A134	3	0	0	0	2	0	0	0	2	9
**2**	A135	0	2	0	0	0	2	0	0	2	9
**2**	A136	1	0	0	0	0	0	0	0	0	0
**2**	A137	0	0	0	0	0	0	0	0	0	0
**2**	A138	0	1	0	0	0	1	0	0	1	4
**2**	A139	0	4	1	0	0	4	1	0	5	22
**2**	A140	1	1	0	1	0	1	0	1	2	9
**2**	A141	5	1	1	0	4	1	1	0	6	26
**2**	A142	1	1	0	0	0	1	0	0	1	4
**2**	A143	0	0	0	0	0	0	0	0	0	0
**2**	A144	2	2	0	0	1	2	0	0	3	13
**2**	A145	16	6	0	0	15	6	0	0	21	91
**2**	A146	1	0	0	0	0	0	0	0	0	0
**2**	A147	0	0	0	0	0	0	0	0	0	0
**2**	A148	3	0	0	0	2	0	0	0	2	9
**2**	A149	3	1	0	0	2	1	0	0	3	13
**2**	A150	6	3	0	0	5	3	0	0	8	35
**2**	A151	3	4	1	1	2	4	1	1	8	35
**2**	A152	24	16	4	0	23	16	4	0	43	187
**2**	A153	0	1	0	0	0	1	0	0	1	4
**2**	A154	1	1	0	0	0	1	0	0	1	4
**2**	A155	2	3	2	0	1	3	2	0	6	26
**2**	A156	0	0	0	0	0	0	0	0	0	0
**2**	A157	0	0	0	0	0	0	0	0	0	0
**2**	A158	0	0	0	0	0	0	0	0	0	0
**2**	A159	7	11	3	0	6	11	3	0	20	87
**2**	A160	0	1	0	0	0	1	0	0	1	4
**2**	A161	0	0	0	0	0	0	0	0	0	0
**2**	A162	0	0	0	0	0	0	0	0	0	0
**2**	A163	0	0	0	0	0	0	0	0	0	0
**2**	A164	4	2	1	0	3	2	1	0	6	26
**2**	A165	1	0	0	0	0	0	0	0	0	0
**2**	A166	0	0	0	0	0	0	0	0	0	0
**2**	A167	0	0	0	0	0	0	0	0	0	0

**2**	A168	1	1	0	0	0	1	0	0	1	4
**2**	A169	2	3	1	0	1	3	1	0	5	22
**2**	A170	1	5	1	0	0	5	1	0	6	26
**2**	A171	1	0	0	0	0	0	0	0	0	0
**2**	A172	0	0	0	0	0	0	0	0	0	0
**2**	A173	0	0	0	0	0	0	0	0	0	0
**2**	A174	1	1	0	0	0	1	0	0	1	4
**2**	A175	2	2	1	0	1	2	1	0	4	17
**2**	A176	0	0	1	0	0	0	1	0	1	4
**2**	A177	0	0	0	0	0	0	0	0	0	0
**2**	A178	0	4	1	0	0	4	1	0	5	22
**2**	A179	7	0	0	0	6	0	0	0	6	26
**2**	A180	1	5	2	0	0	5	2	0	7	30
**2**	A181	0	0	0	0	0	0	0	0	0	0
**2**	A182	1	0	0	0	0	0	0	0	0	0
**2**	A183	-	-	-	-	-	-	-	-	-	-
**2**	A184	0	0	0	0	0	0	0	0	0	0
**2**	A185	3	0	0	0	2	0	0	0	2	9
**2**	A186	1	0	0	0	0	0	0	0	0	0
**2**	A187	0	0	0	0	0	0	0	0	0	0
**2**	A188	0	0	0	0	0	0	0	0	0	0
**2**	A189	2	0	1	0	1	0	1	0	2	9
**2**	A190	22	2	0	0	21	2	0	0	23	100
**2**	A191	2	0	0	0	1	0	0	0	1	4
**2**	A192	1	2	0	0	0	2	0	0	2	9
**2**	A193	1	0	0	0	0	0	0	0	0	0
**2**	A194	2	0	0	0	1	0	0	0	1	4
**2**	A195	0	0	0	0	0	0	0	0	0	0
**2**	A196	2	1	0	0	1	1	0	0	2	9
**2**	A197	0	0	0	0	0	0	0	0	0	0
**2**	A198	0	0	0	0	0	0	0	0	0	0
**2**	A199	1	0	0	0	0	0	0	0	0	0
**2**	A200	1	0	0	0	0	0	0	0	0	0
**2**	A201	0	0	0	0	0	0	0	0	0	0
**2**	A202	0	1	0	0	0	1	0	0	1	4
**2**	A203	2	3	5	0	1	3	5	0	9	39
**2**	A204	0	1	0	0	0	1	0	0	1	4
**2**	A205	3	2	0	2	2	2	0	2	6	26
**2**	A206	1	0	0	1	0	0	0	1	1	4
**2**	A207	2	0	0	0	1	0	0	0	1	4
**2**	A208	2	1	0	0	1	1	0	0	2	9
**2**	A209	0	0	0	0	0	0	0	0	0	0
**2**	A210	1	0	0	0	0	0	0	0	0	0
**2**	A211	0	0	0	0	0	0	0	0	0	0
**2**	A212	4	0	0	0	3	0	0	0	3	13
**2**	A213	0	0	0	0	0	0	0	0	0	0
**2**	A214	0	3	2	0	0	3	2	0	5	22
**2**	A215	0	3	2	0	0	3	2	0	5	22

**2**	A216	1	1	0	0	0	1	0	0	1	4
**2**	A217	0	0	2	0	0	0	2	0	2	9
**2**	A218	2	1	0	0	1	1	0	0	2	9
**2**	A219	0	0	0	0	0	0	0	0	0	0
**2**	A220	0	0	0	1	0	0	0	1	1	4
**2**	A221	0	0	0	0	0	0	0	0	0	0
**2**	A222	0	0	0	0	0	0	0	0	0	0
**2**	A223	1	2	0	0	0	2	0	0	2	9
**2**	A224	0	0	0	0	0	0	0	0	0	0
**2**	A225	0	0	0	0	0	0	0	0	0	0
**2**	A226	0	0	0	0	0	0	0	0	0	0
**2**	A227	0	1	0	0	0	1	0	0	1	4
**2**	A228	0	0	0	0	0	0	0	0	0	0
**2**	A229	0	0	0	0	0	0	0	0	0	0
**2**	A230	0	2	1	0	0	2	1	0	3	13
**2**	A231	0	0	1	0	0	0	1	0	1	4
**2**	A232	1	0	0	0	0	0	0	0	0	0
**2**	A233	0	0	0	0	0	0	0	0	0	0
**2**	A234	0	0	0	0	0	0	0	0	0	0
**2**	A235	0	1	0	0	0	1	0	0	1	4
**2**	A236	0	0	1	0	0	0	1	0	1	4
**2**	A237	0	0	0	0	0	0	0	0	0	0
**2**	A238	0	0	0	0	0	0	0	0	0	0
**2**	A239	1	0	0	0	0	0	0	0	0	0
**2**	A240	0	0	0	0	0	0	0	0	0	0
**2**	A241	0	1	0	0	0	1	0	0	1	4
**2**	A242	0	0	0	0	0	0	0	0	0	0
**2**	A243	0	0	0	0	0	0	0	0	0	0
**2**	A244	0	0	0	0	0	0	0	0	0	0
**2**	A245	0	0	0	0	0	0	0	0	0	0
**2**	A246	0	0	0	0	0	0	0	0	0	0
**2**	A247	1	0	0	0	0	0	0	0	0	0

Particle library

Batch 1

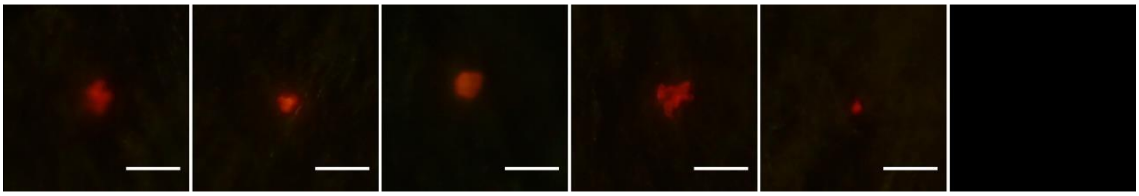

Figure S4. Fluorescent particles (suspected microplastics), with a 50 µm scale, observed in 23% of the filter (without blank subtraction) of A001.

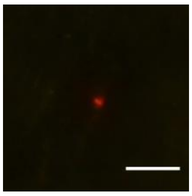

Figure S5. Fluorescent particles (suspected microplastics), with a 50 µm scale, observed in 23% of the filter (without blank subtraction) of A002.

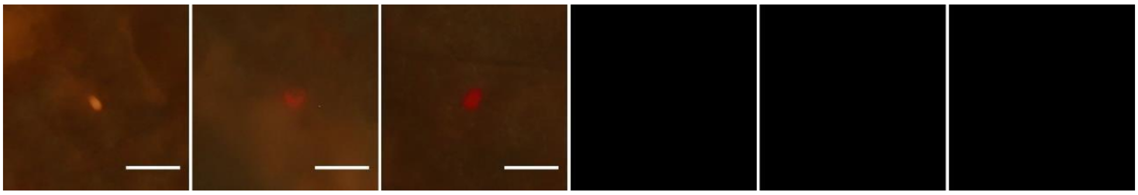

Figure S6. Fluorescent particles (suspected microplastics), with a 50 µm scale, observed in 23% of the filter (without blank subtraction) of A003.

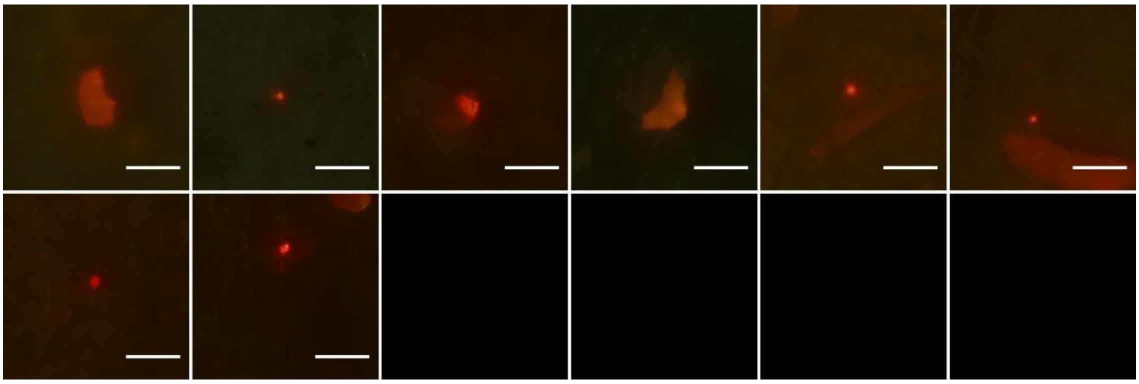

Figure S7. Fluorescent particles (suspected microplastics), with a 50 µm scale, observed in 23% of the filter (without blank subtraction) of A004.

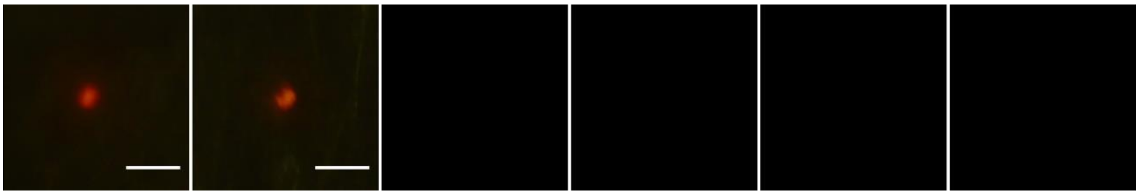

Figure S8. Fluorescent particles (suspected microplastics), with a 50 µm scale, observed in 23% of the filter (without blank subtraction) of A005.

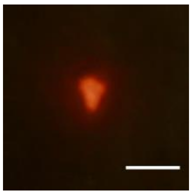

Figure S9. Fluorescent particles (suspected microplastics), with a 50 µm scale, observed in 23% of the filter (without blank subtraction) of A007.

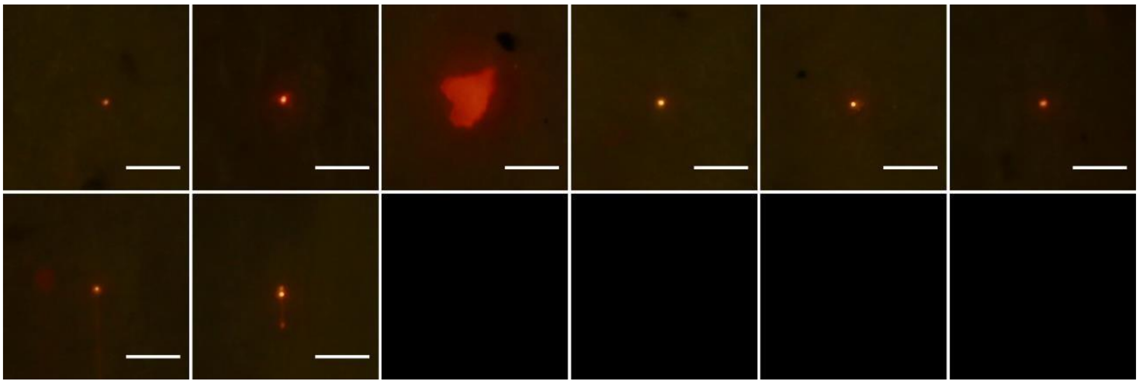

Figure S10. Fluorescent particles (suspected microplastics), with a 50 µm scale, observed in 23% of the filter (without blank subtraction) of A008.

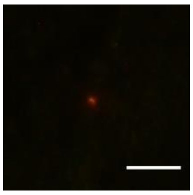

Figure S11. Fluorescent particles (suspected microplastics), with a 50 µm scale, observed in 23% of the filter (without blank subtraction) of A009.

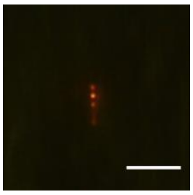

Figure S12. Fluorescent particles (suspected microplastics), with a 50 µm scale, observed in 23% of the filter (without blank subtraction) of A010.

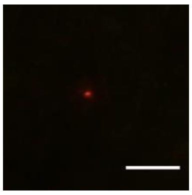

Figure S13. Fluorescent particles (suspected microplastics), with a 50 µm scale, observed in 23% of the filter (without blank subtraction) of A011.

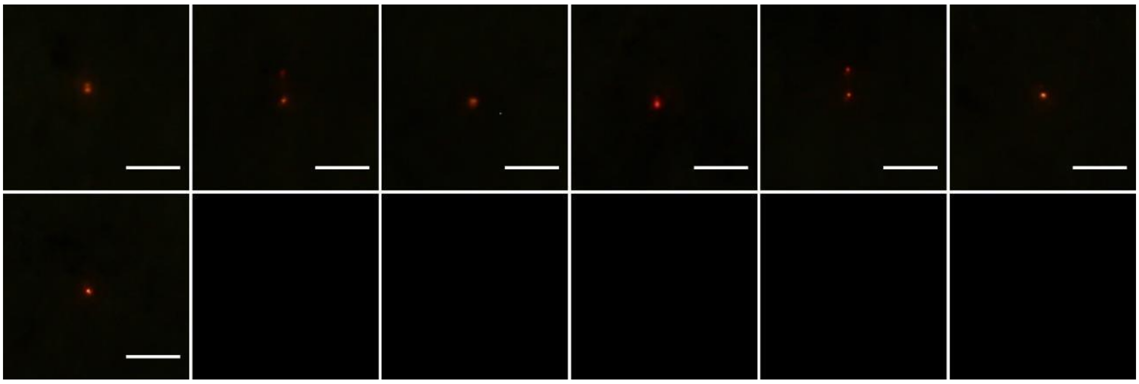

Figure S14. Fluorescent particles (suspected microplastics), with a 50 µm scale, observed in 23% of the filter (without blank subtraction) of A012.

Figure S15. Fluorescent particles (suspected microplastics), with a 50 µm scale, observed in 23% of the filter (without blank subtraction) of A015.

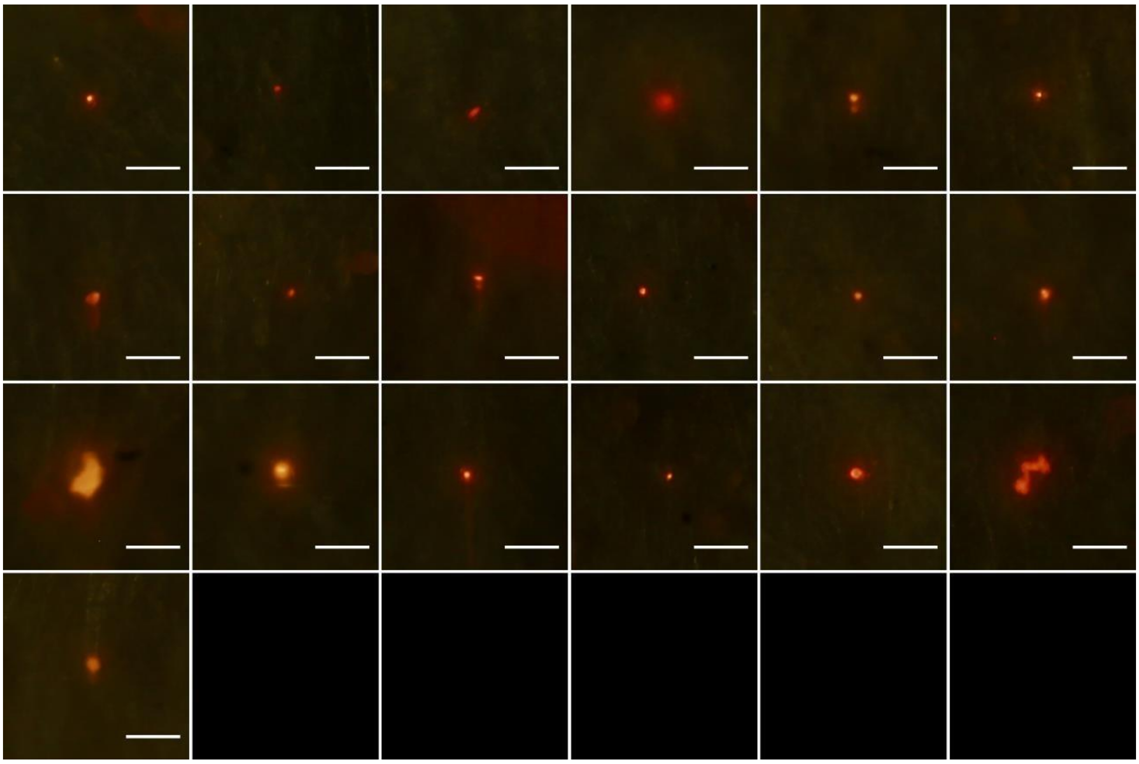

Figure S16. Fluorescent particles (suspected microplastics), with a 50 µm scale, observed in 23% of the filter (without blank subtraction) of A017.

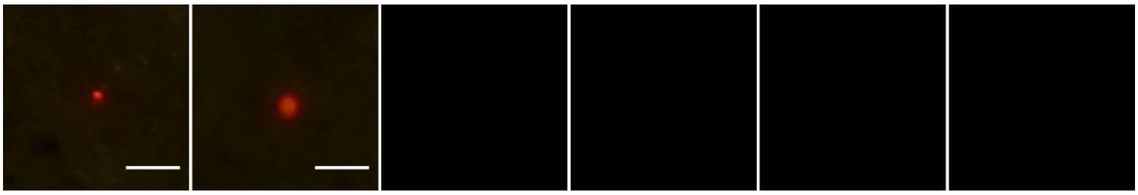

Figure S17. Fluorescent particles (suspected microplastics), with a 50 µm scale, observed in 23% of the filter (without blank subtraction) of A018.

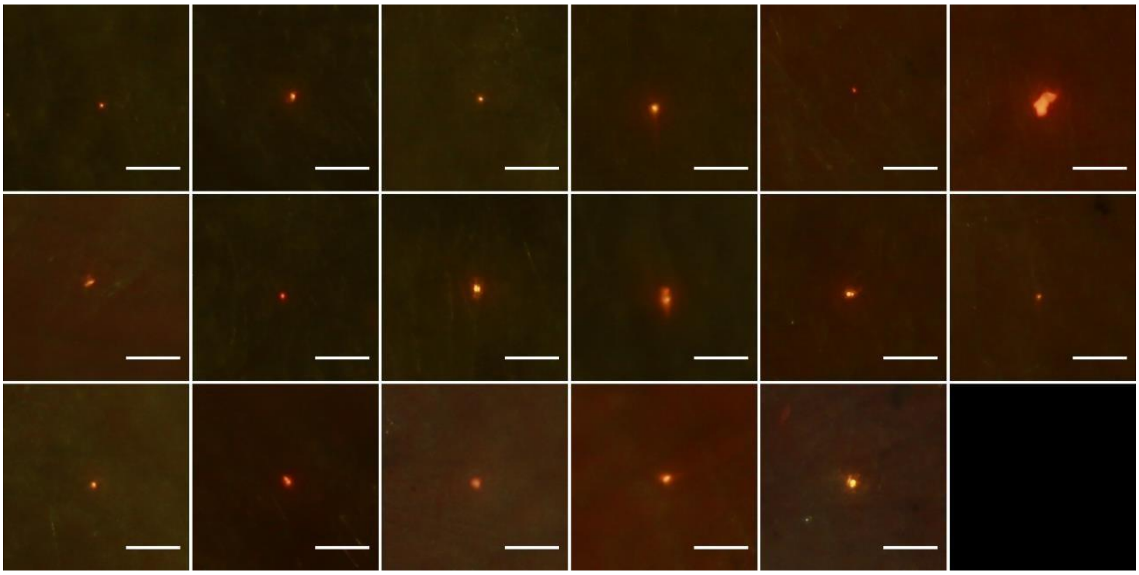

Figure S18. Fluorescent particles (suspected microplastics), with a 50 µm scale, observed in 23% of the filter (without blank subtraction) of A019.

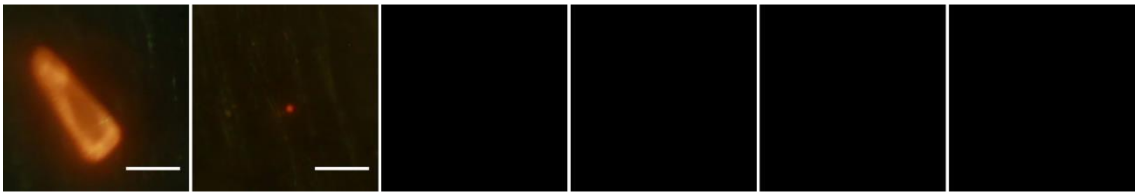

Figure S19. Fluorescent particles (suspected microplastics), with a 50 µm scale, observed in 23% of the filter (without blank subtraction) of A020.

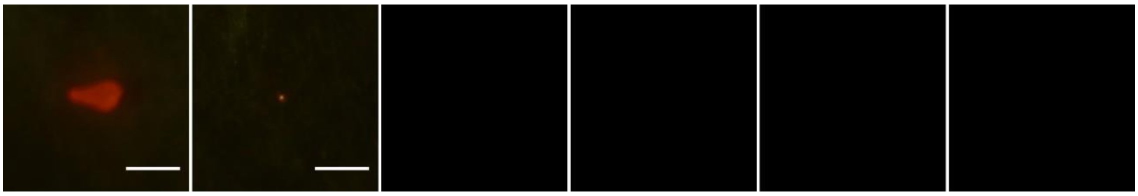

Figure S20. Fluorescent particles (suspected microplastics), with a 50 µm scale, observed in 23% of the filter (without blank subtraction) of A021.

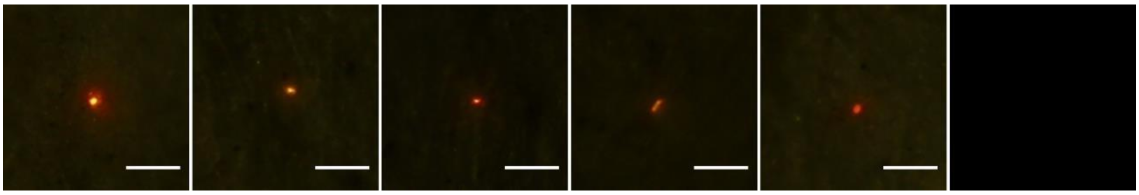

Figure S21. Fluorescent particles (suspected microplastics), with a 50 µm scale, observed in 23%
of the filter (without blank subtraction) of A022.
the

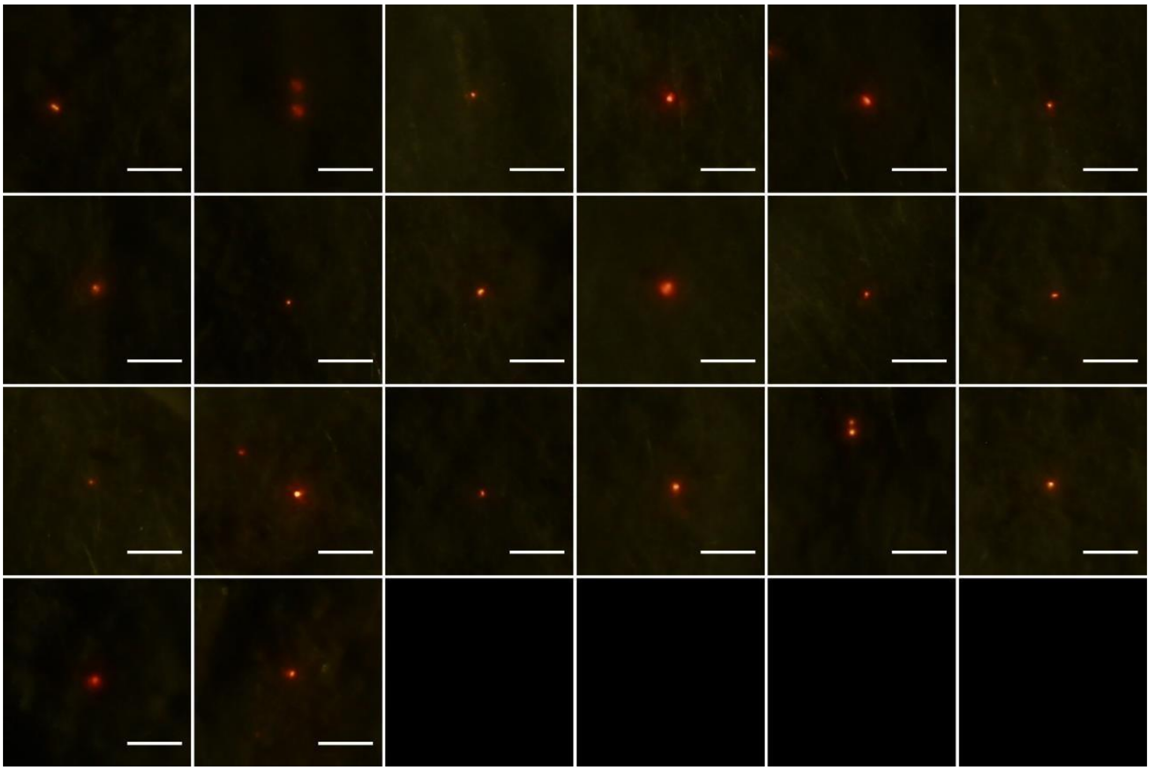

Figure S22. Fluorescent particles (suspected microplastics), with a 50 µm scale, observed in 23% of the filter (without blank subtraction) of A023.

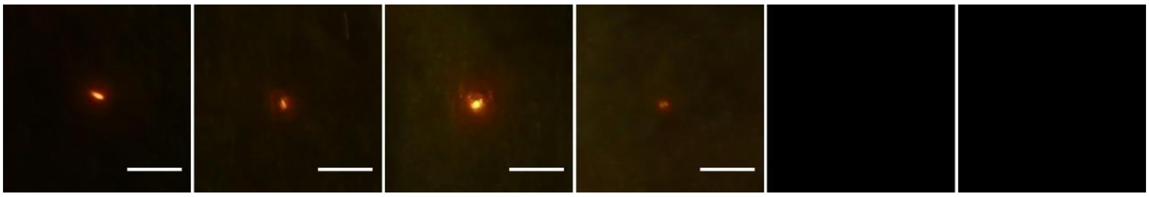

Figure S23. Fluorescent particles (suspected microplastics), with a 50 µm scale, observed in 23% of the filter (without blank subtraction) of A024.

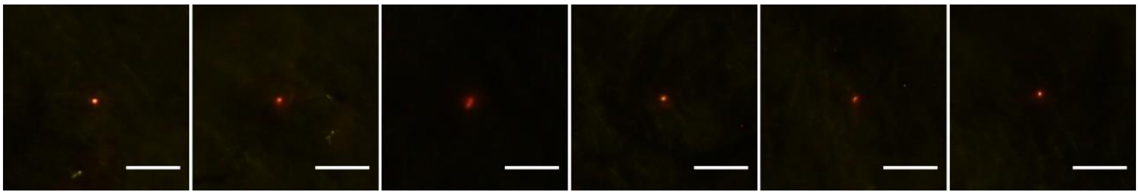

Figure S24. Fluorescent particles (suspected microplastics), with a 50 µm scale, observed in 23% of the filter (without blank subtraction) of A026.

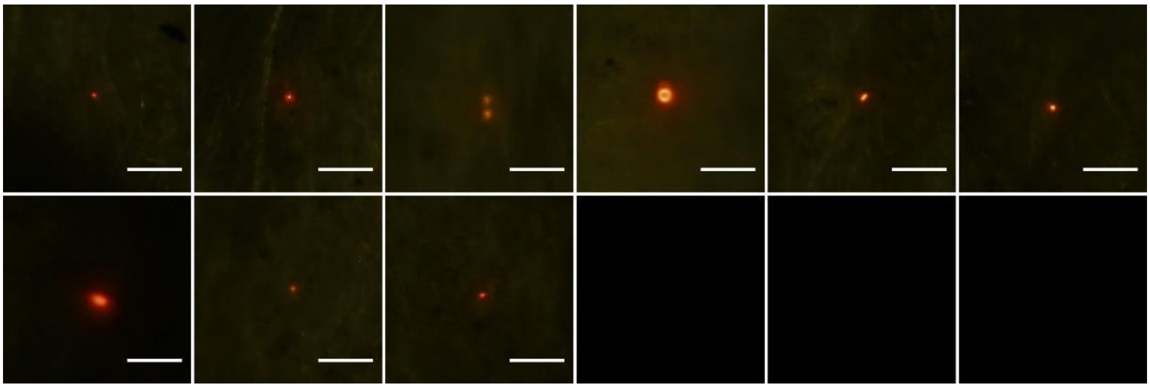

Figure S25. Fluorescent particles (suspected microplastics), with a 50 µm scale, observed in 23% of the filter (without blank subtraction) of A027.

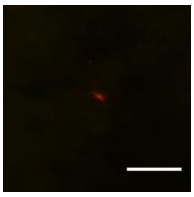

Figure S26. Fluorescent particles (suspected microplastics), with a 50 µm scale, observed in 23% of the filter (without blank subtraction) of A028.

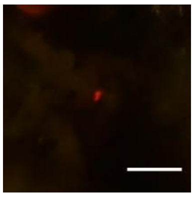

Figure S27. Fluorescent particles (suspected microplastics), with a 50 µm scale, observed in 23% of the filter (without blank subtraction) of A029.

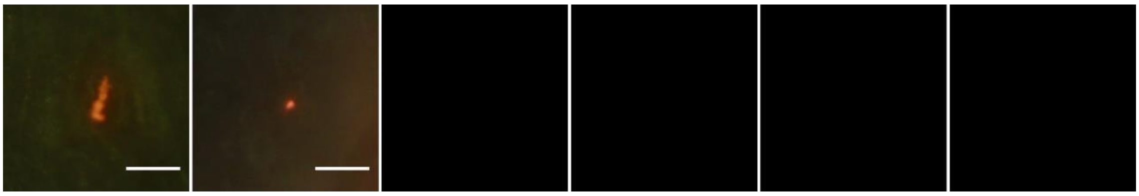

Figure S28. Fluorescent particles (suspected microplastics), with a 50 µm scale, observed in 23% of the filter (without blank subtraction) of A032.

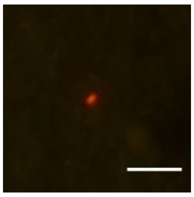

Figure S29. Fluorescent particles (suspected microplastics), with a 50 µm scale, observed in 23% of the filter (without blank subtraction) of A033.

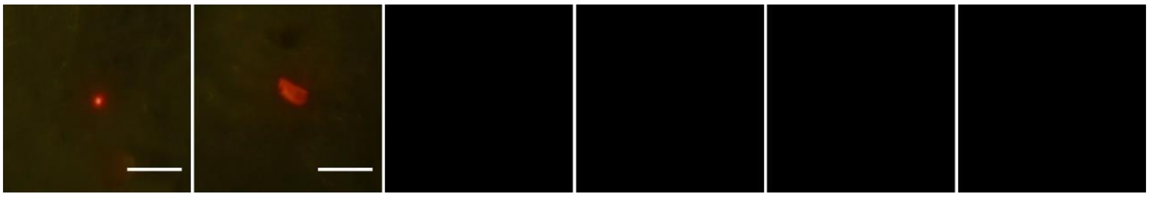

Figure S30. Fluorescent particles (suspected microplastics), with a 50 µm scale, observed in 23% of the filter (without blank subtraction) of A037.

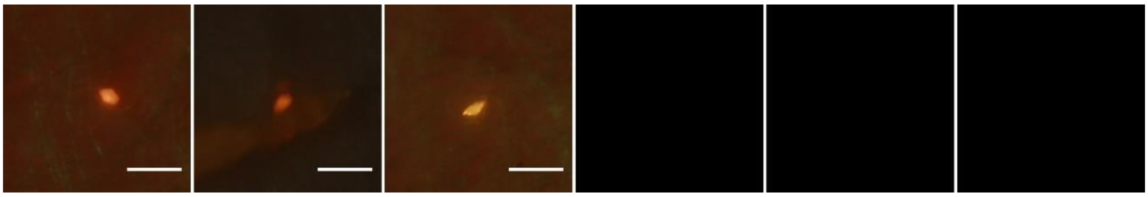

Figure S31. Fluorescent particles (suspected microplastics), with a 50 µm scale, observed in 23% of the filter (without blank subtraction) of A038.

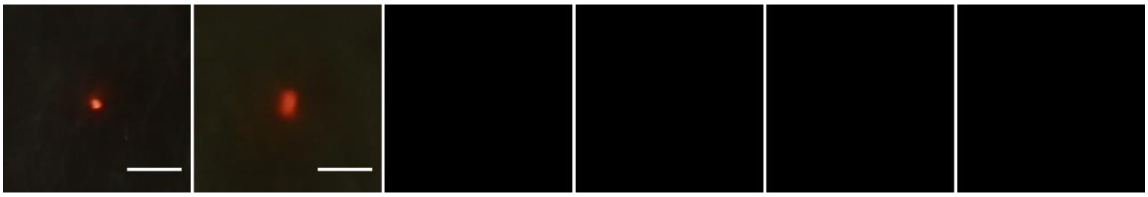

Figure S32. Fluorescent particles (suspected microplastics), with a 50 µm scale, observed in 23% of the filter (without blank subtraction) of A040.

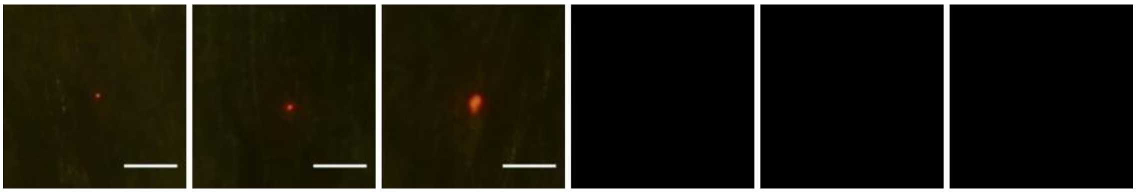

Figure S33. Fluorescent particles (suspected microplastics), with a 50 µm scale, observed in 23% of the filter (without blank subtraction) of A041.

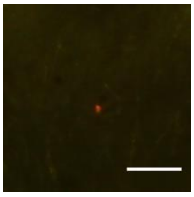

Figure S34. Fluorescent particles (suspected microplastics), with a 50 µm scale, observed in 23% of the filter (without blank subtraction) of A042.

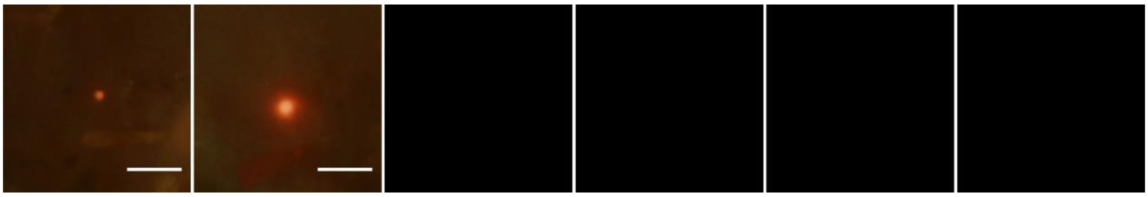

Figure S35. Fluorescent particles (suspected microplastics), with a 50 µm scale, observed in 23% of the filter (without blank subtraction) of A044.

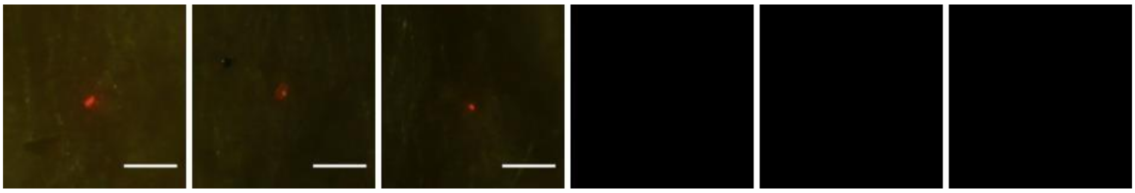

Figure S36. Fluorescent particles (suspected microplastics), with a 50 µm scale, observed in 23% of the filter (without blank subtraction) of A046.

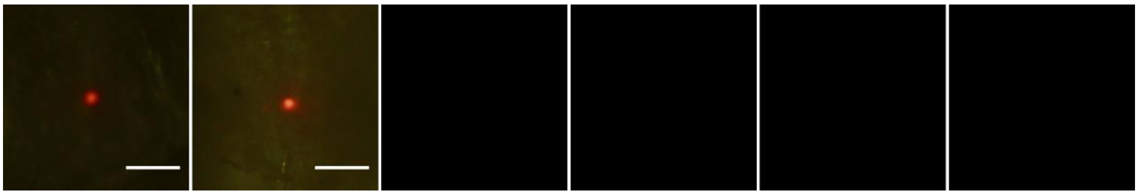

Figure S37. Fluorescent particles (suspected microplastics), with a 50 µm scale, observed in 23% of the filter (without blank subtraction) of A047.

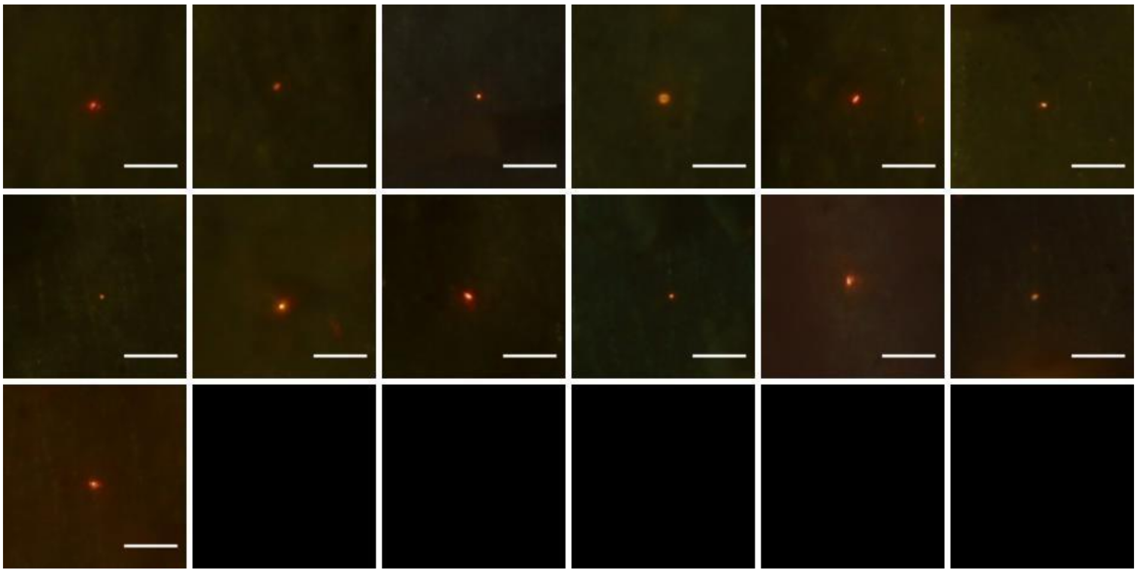

Figure S38. Fluorescent particles (suspected microplastics), with a 50 µm scale, observed in 23% of the filter (without blank subtraction) of A048.

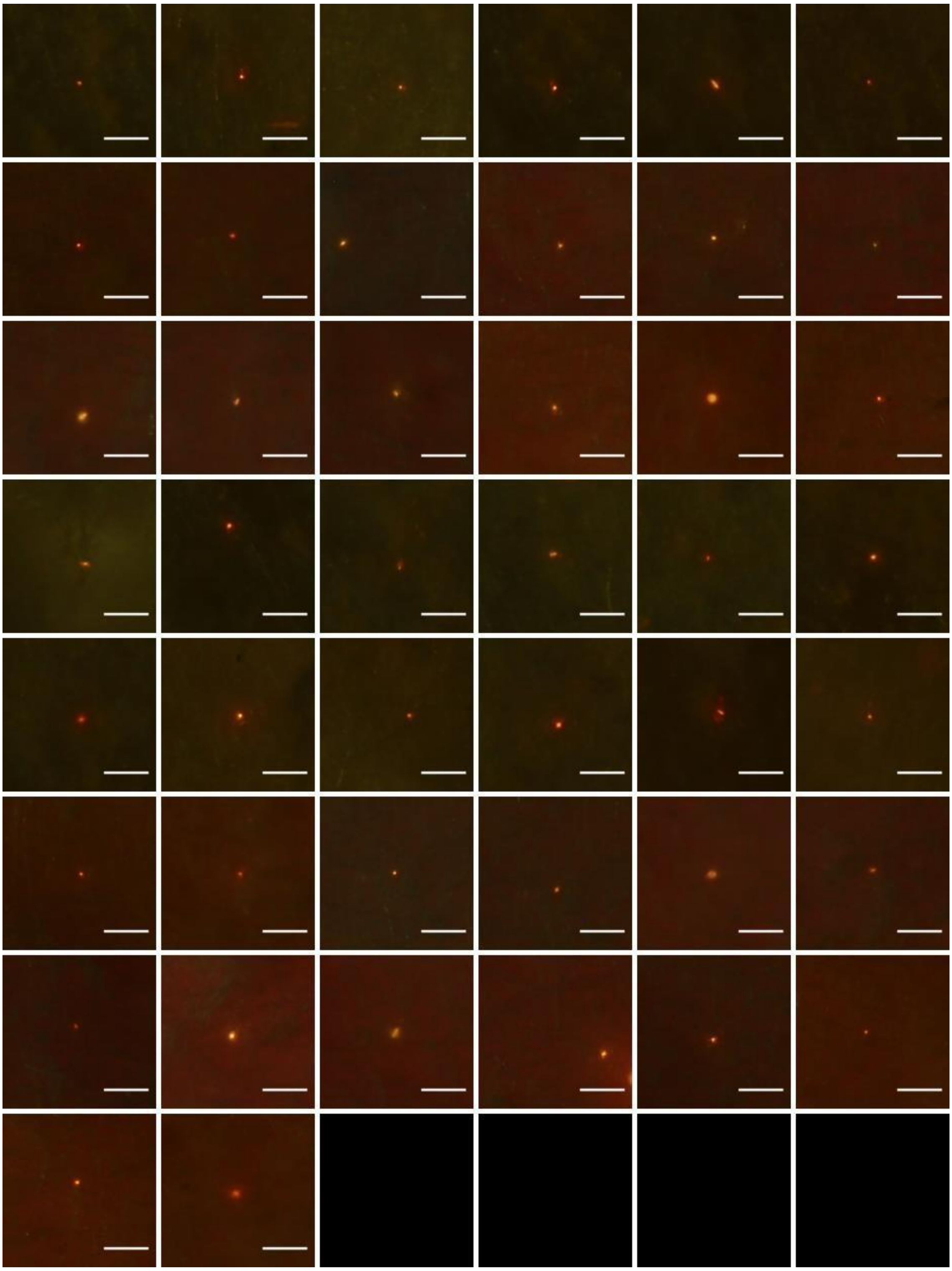

Figure S39. Fluorescent particles (suspected microplastics), with a 50 µm scale, observed in 23% of the filter (without blank subtraction) of A049.

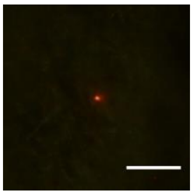

Figure S40. Fluorescent particles (suspected microplastics), with a 50 µm scale, observed in 23% of the filter (without blank subtraction) of A051.

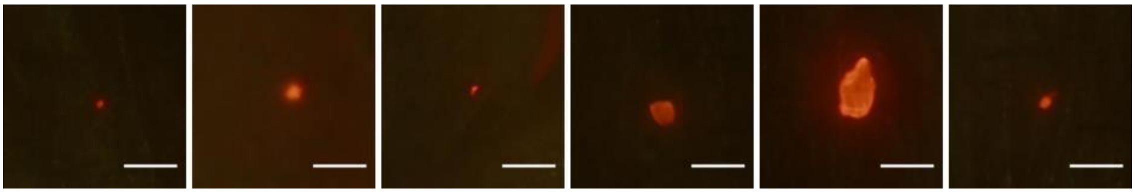

Figure S41. Fluorescent particles (suspected microplastics), with a 50 µm scale, observed in 23% of the filter (without blank subtraction) of A052.

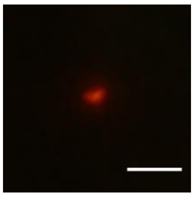

Figure S42. Fluorescent particles (suspected microplastics), with a 50 µm scale, observed in 23% of the filter (without blank subtraction) of A055.

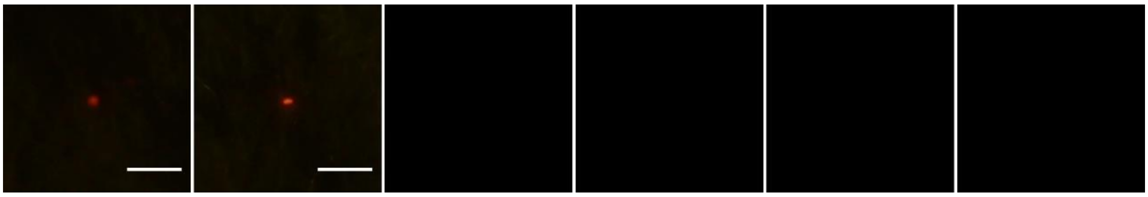

Figure S43. Fluorescent particles (suspected microplastics), with a 50 µm scale, observed in 23% of the filter (without blank subtraction) of A056.

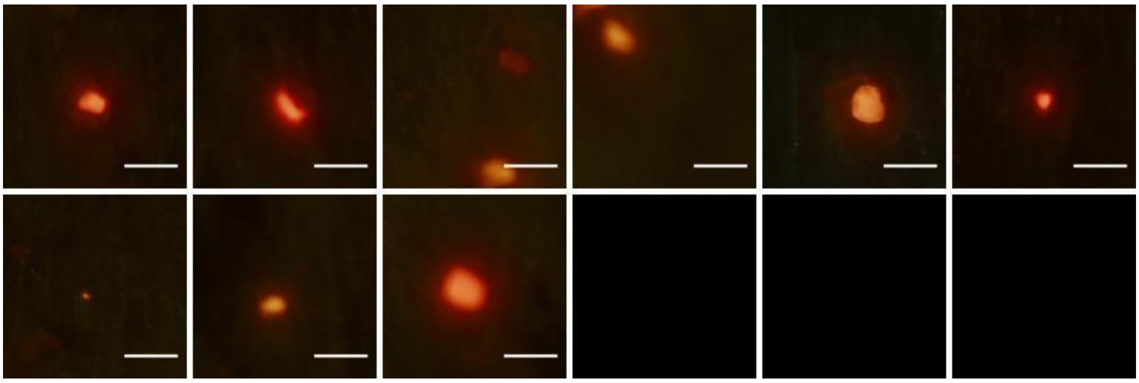

Figure S44. Fluorescent particles (suspected microplastics), with a 50 µm scale, observed in 23% of the filter (without blank subtraction) of A058.

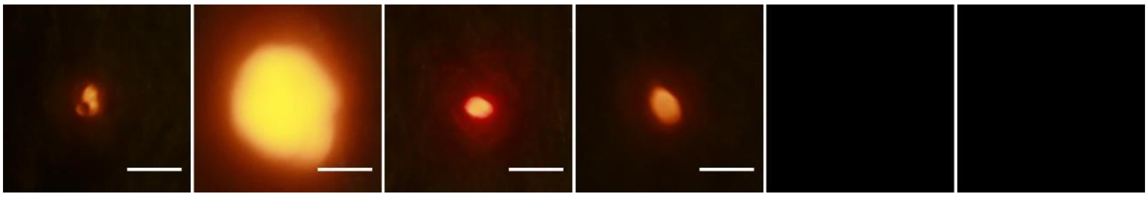

Figure S45. Fluorescent particles (suspected microplastics), with a 50 µm scale, observed in 23% of the filter (without blank subtraction) of A059.

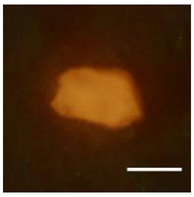

Figure S46. Fluorescent particles (suspected microplastics), with a 50 µm scale, observed in 23% of the filter (without blank subtraction) of A069.

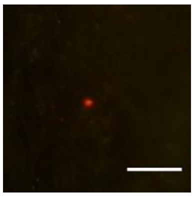

Figure S47. Fluorescent particles (suspected microplastics), with a 50 µm scale, observed in 23% of the filter (without blank subtraction) of A071.

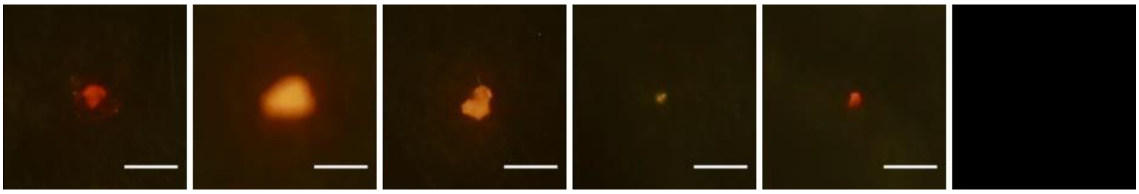

Figure S48. Fluorescent particles (suspected microplastics), with a 50 µm scale, observed in 23% of the filter (without blank subtraction) of A072.

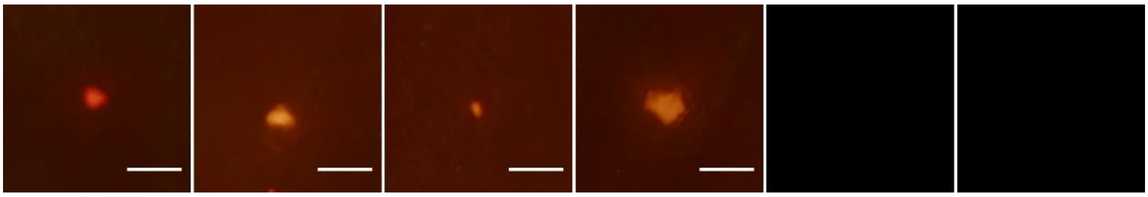

Figure S49. Fluorescent particles (suspected microplastics), with a 50 µm scale, observed in 23% of the filter (without blank subtraction) of A073.

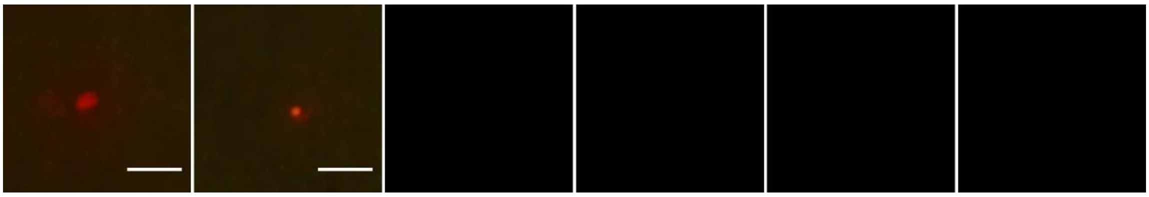

Figure S50. Fluorescent particles (suspected microplastics), with a 50 µm scale, observed in 23% of the filter (without blank subtraction) of A074.

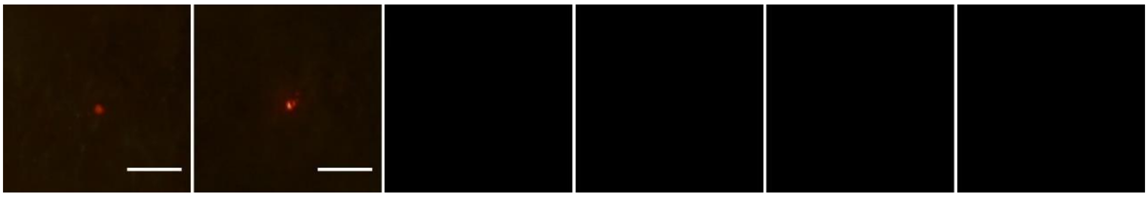

Figure S51. Fluorescent particles (suspected microplastics), with a 50 µm scale, observed in 23% of the filter (without blank subtraction) of A077.

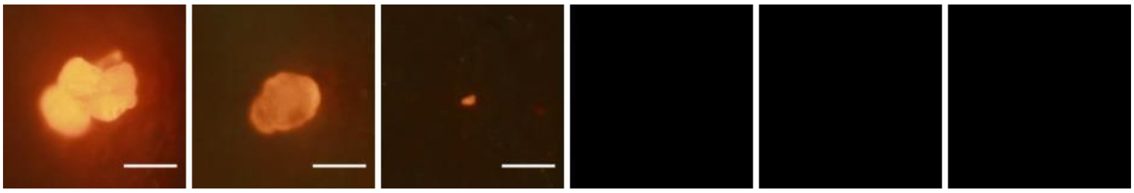

Figure S52. Fluorescent particles (suspected microplastics), with a 50 µm scale, observed in 23% of the filter (without blank subtraction) of A079.

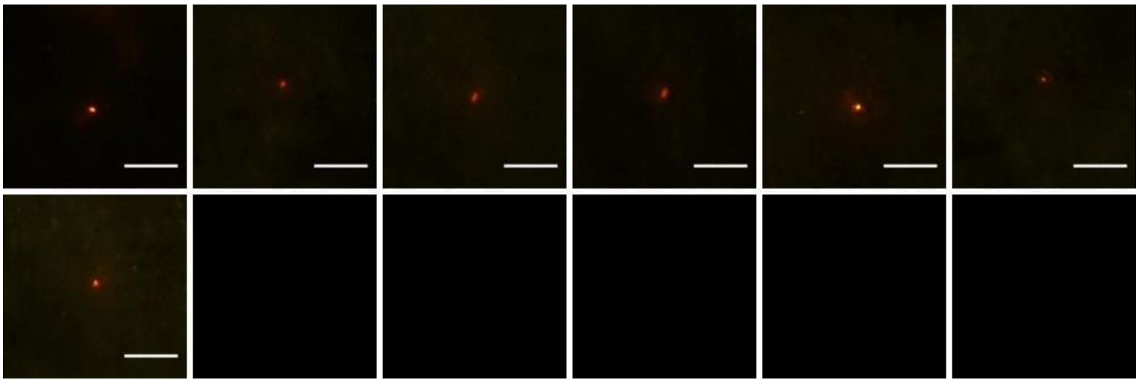

Figure S53. Fluorescent particles (suspected microplastics), with a 50 µm scale, observed in 23% of the filter (without blank subtraction) of A082.

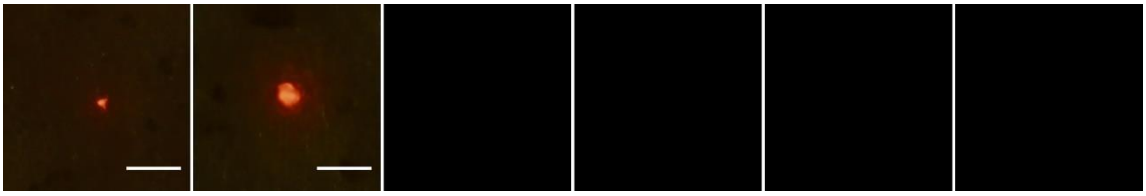

Figure S54. Fluorescent particles (suspected microplastics), with a 50 µm scale, observed in 23% of the filter (without blank subtraction) of A084.

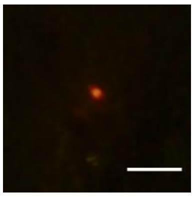

Figure S55. Fluorescent particles (suspected microplastics), with a 50 µm scale, observed in 23% of the filter (without blank subtraction) of A086.

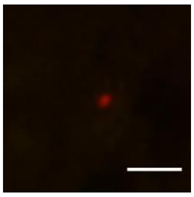

Figure S56. Fluorescent particles (suspected microplastics), with a 50 µm scale, observed in 23% of the filter (without blank subtraction) of A087.

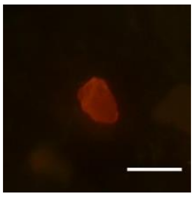

Figure S57. Fluorescent particles (suspected microplastics), with a 50 µm scale, observed in 23% of the filter (without blank subtraction) of A088.

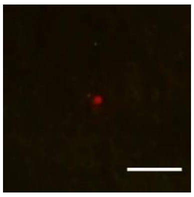

Figure S58. Fluorescent particles (suspected microplastics), with a 50 µm scale, observed in 23% of the filter (without blank subtraction) of A089.

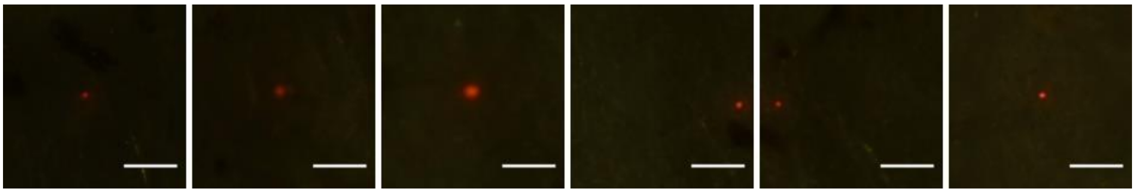

Figure S59. Fluorescent particles (suspected microplastics), with a 50 µm scale, observed in 23% of the filter (without blank subtraction) of A092.

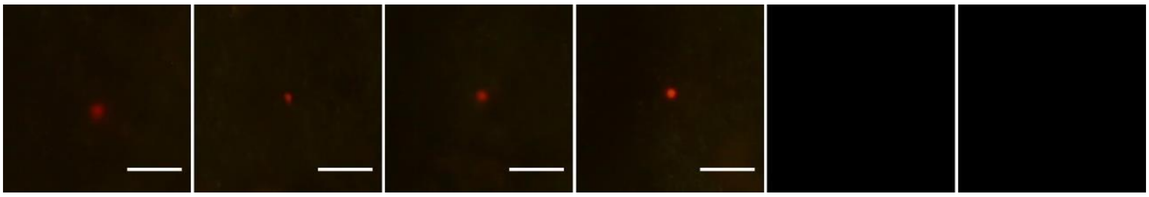

Figure S60. Fluorescent particles (suspected microplastics), with a 50 µm scale, observed in 23% of the filter (without blank subtraction) of A093.

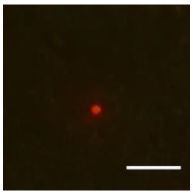

Figure S61. Fluorescent particles (suspected microplastics), with a 50 µm scale, observed in 23 of the filter (without blank subtraction) of A094.

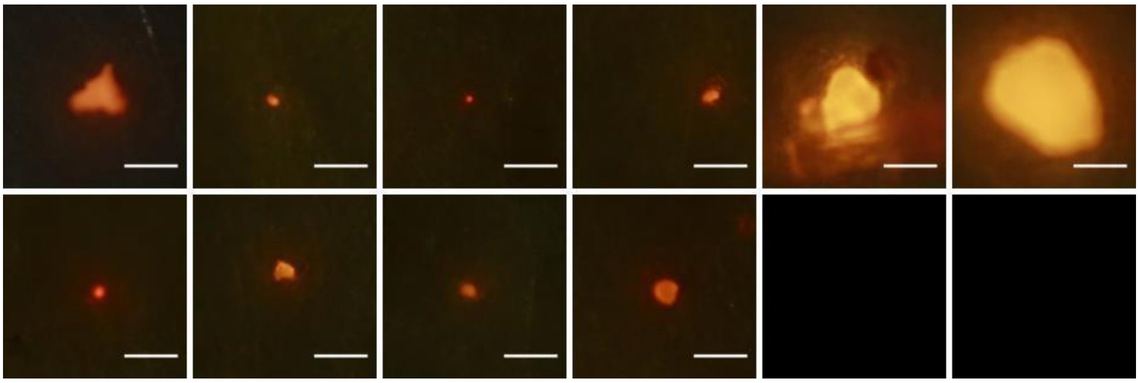

Figure S62. Fluorescent particles (suspected microplastics), with a 50 µm scale, observed in 23% of the filter (without blank subtraction) of A095.

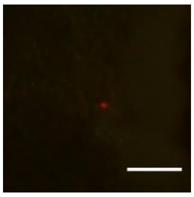

Figure S63. Fluorescent particles (suspected microplastics), with a 50 µm scale, observed in 23% of the filter (without blank subtraction) of A108.

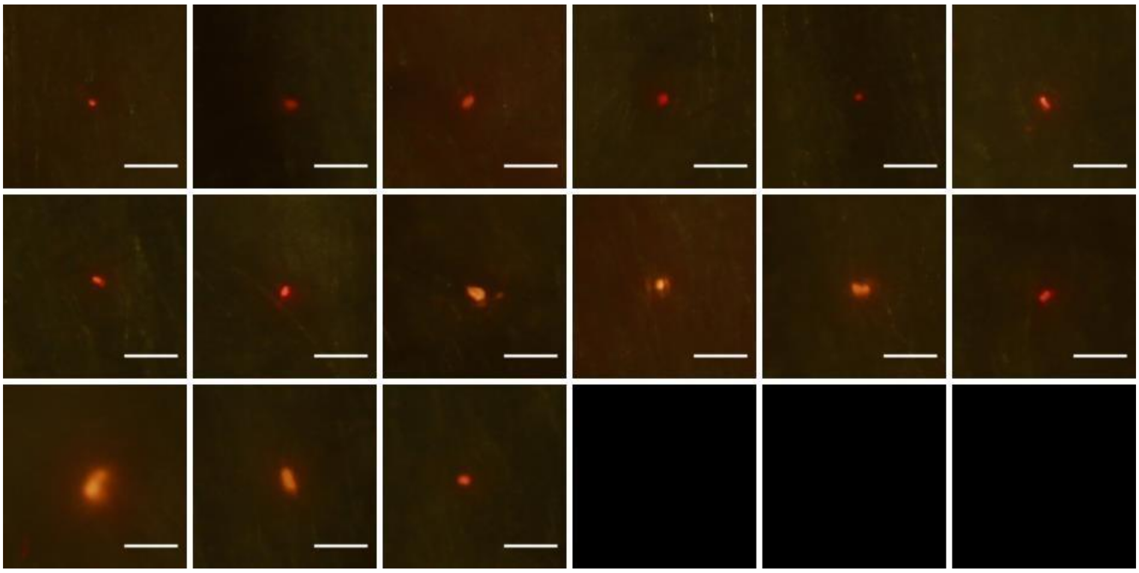

Figure S64. Fluorescent particles (suspected microplastics), with a 50 µm scale, observed in 23%

of the filter of blank 1 (batch 1).

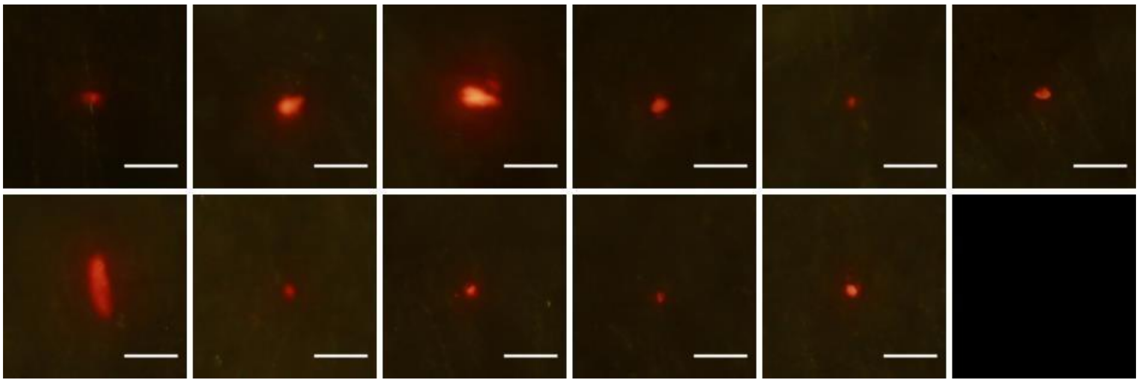

Figure S65. Fluorescent particles (suspected microplastics), with a 50 µm scale, observed in 23% of the filter (without blank subtraction) of blank 2 (batch 1).

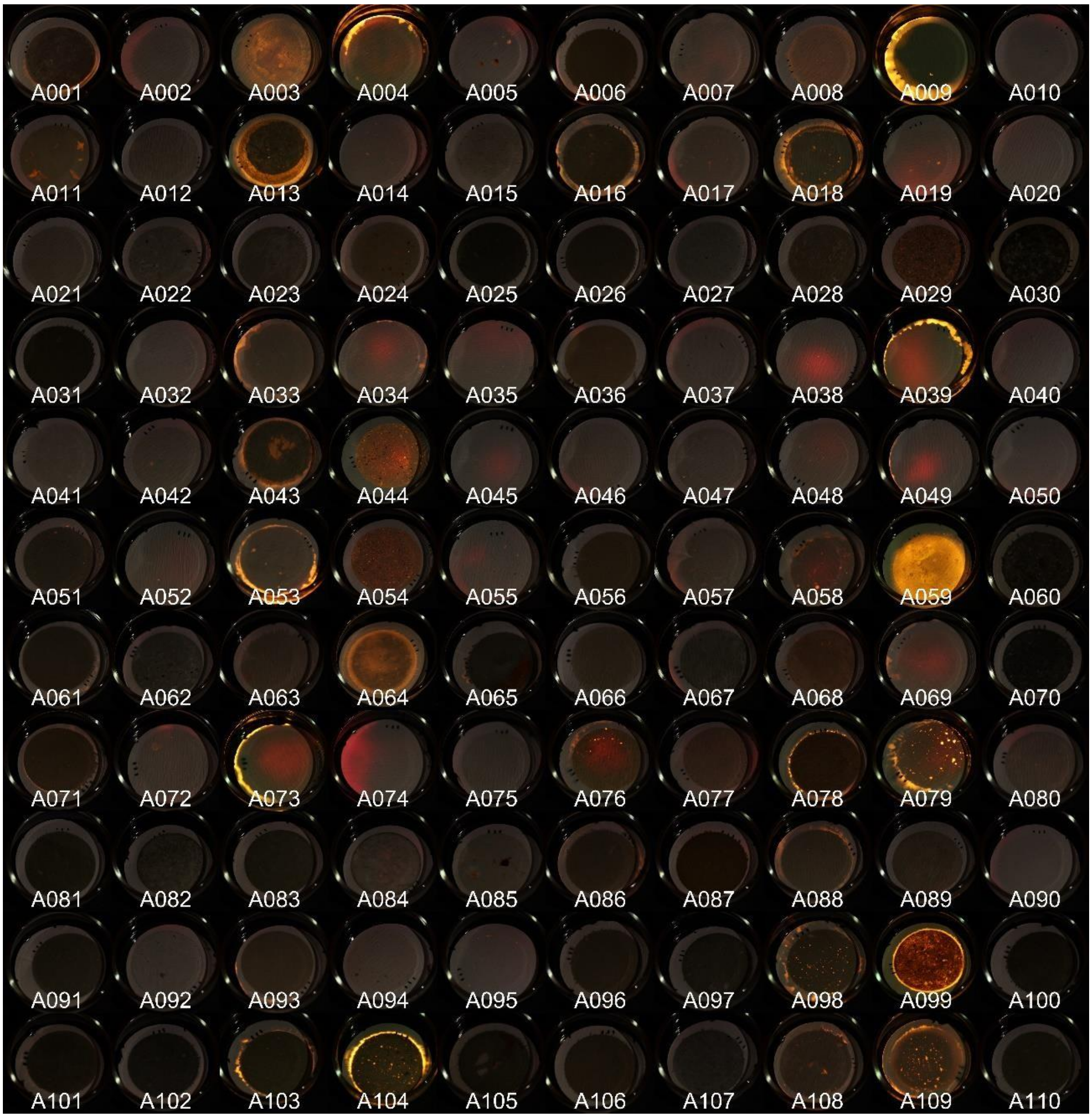

Figure S66. Filter membranes of the batch 1 of companion animal samples.

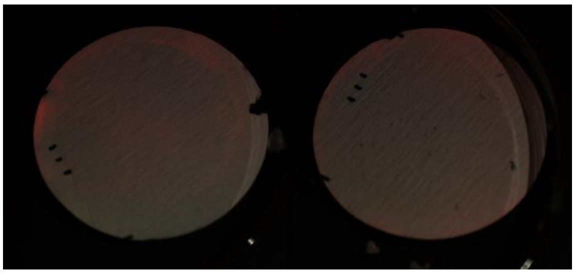

Figure S67. Filter membranes of batch 1 blanks.

Batch 2

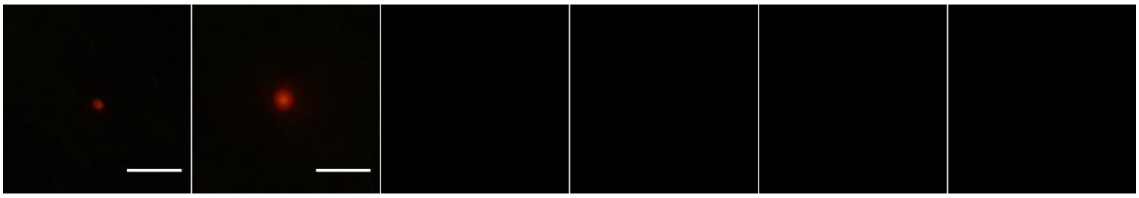

Figure S68. Fluorescent particles (suspected microplastics), with a 50 µm scale, observed in 23% of the filter (without blank subtraction) of A111.

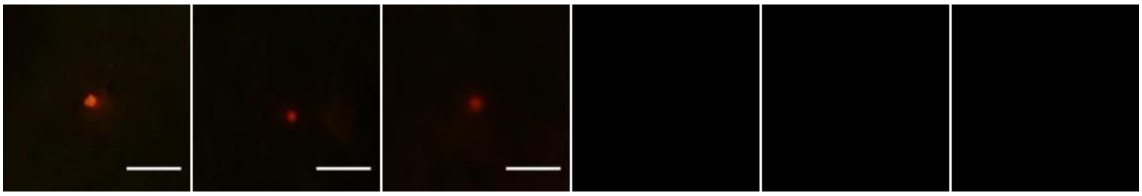

Figure S69. Fluorescent particles (suspected microplastics), with a 50 µm scale, observed in 23% of the filter (without blank subtraction) of A112.

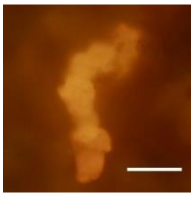

Figure S70. Fluorescent particles (suspected microplastics), with a 50 µm scale, observed in 23% of the filter (without blank subtraction) of A113.

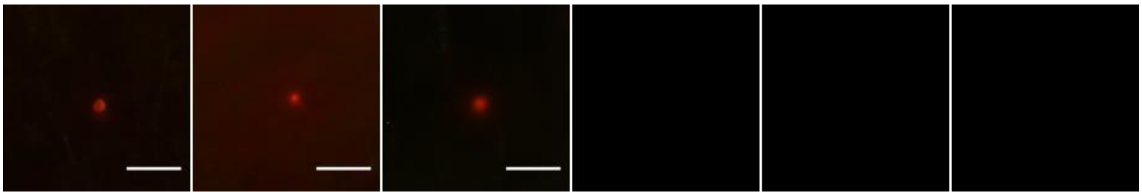

Figure S71. Fluorescent particles (suspected microplastics), with a 50 µm scale, observed in 23% of the filter (without blank subtraction) of A115.

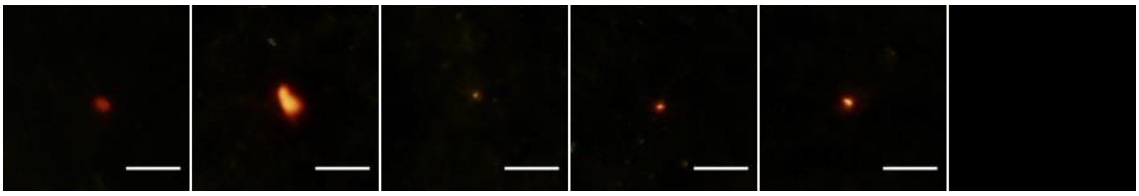

Figure S72. Fluorescent particles (suspected microplastics), with a 50 µm scale, observed in 23% of the filter (without blank subtraction) of A122.

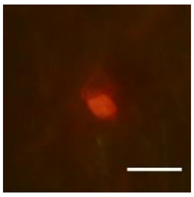

Figure S73. Fluorescent particles (suspected microplastics), with a 50 µm scale, observed in 23% of the filter (without blank subtraction) of A124.

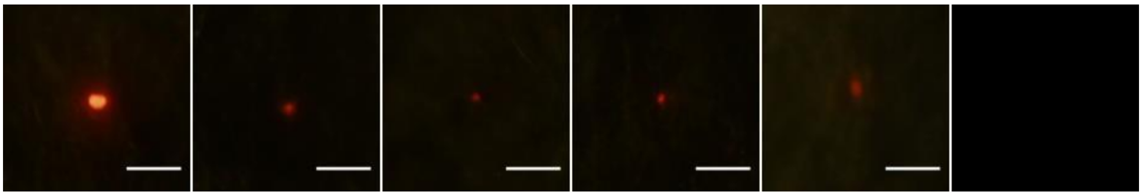

Figure S74. Fluorescent particles (suspected microplastics), with a 50 µm scale, observed in 23% of the filter (without blank subtraction) of A125.

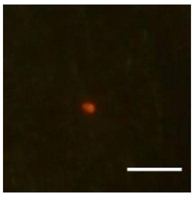

Figure S75. Fluorescent particles (suspected microplastics), with a 50 µm scale, observed in 23% of the filter (without blank subtraction) of A126.

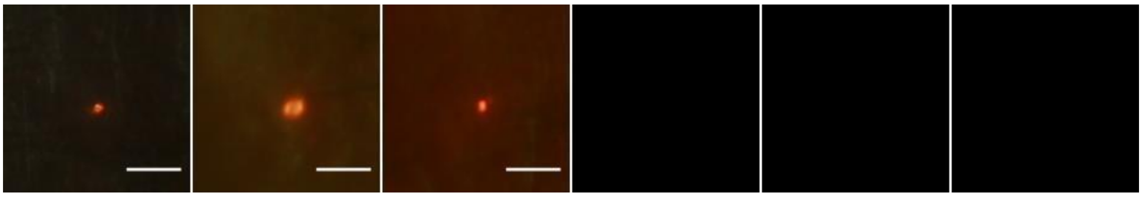

Figure S76. Fluorescent particles (suspected microplastics), with a 50 µm scale, observed in 23% of the filter (without blank subtraction) of A127.

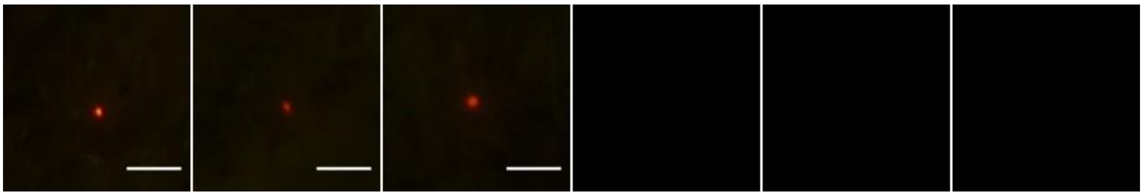

Figure S77. Fluorescent particles (suspected microplastics), with a 50 µm scale, observed in 23% of the filter (without blank subtraction) of A134.

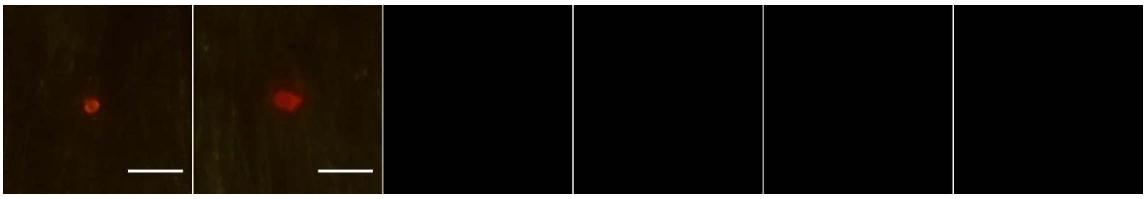

Figure S78. Fluorescent particles (suspected microplastics), with a 50 µm scale, observed in 23% of the filter (without blank subtraction) of A135.

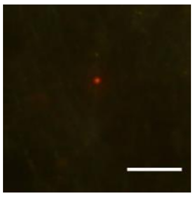

Figure S79. Fluorescent particles (suspected microplastics), with a 50 µm scale, observed in 23% of the filter (without blank subtraction) of A136.

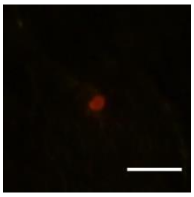

Figure S80. Fluorescent particles (suspected microplastics), with a 50 µm scale, observed in 23% of the filter (without blank subtraction) of A138.

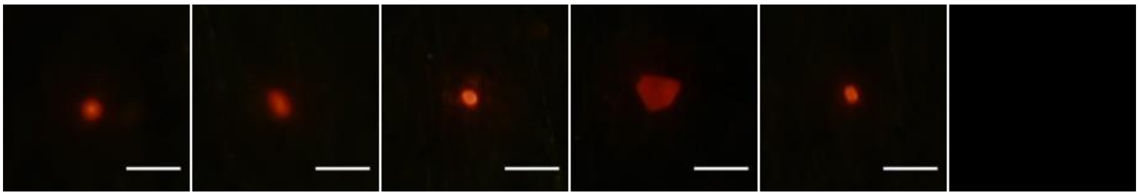

Figure S81. Fluorescent particles (suspected microplastics), with a 50 µm scale, observed in 23% of the filter (without blank subtraction) of A139.

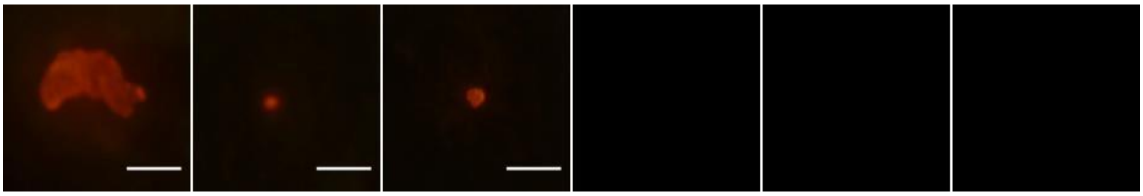

Figure S82. Fluorescent particles (suspected microplastics), with a 50 µm scale, observed in 23% of the filter (without blank subtraction) of A140.

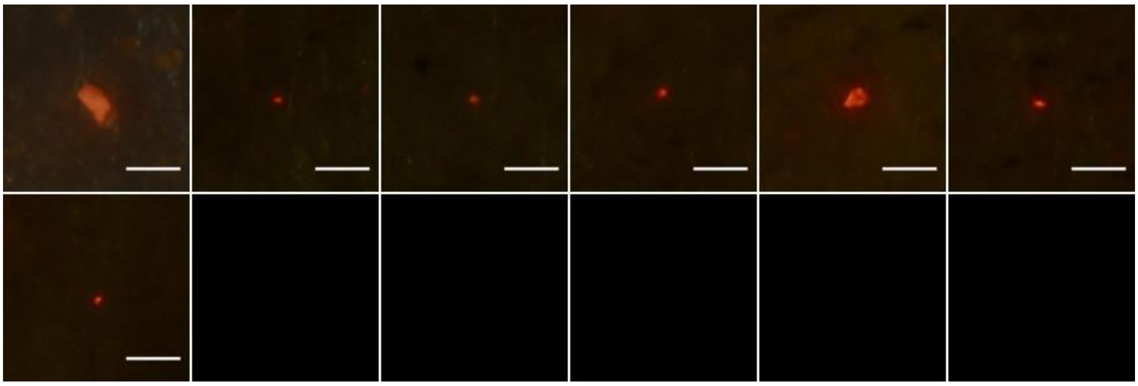

Figure S83. Fluorescent particles (suspected microplastics), with a 50 µm scale, observed in 23% of the filter (without blank subtraction) of A141.

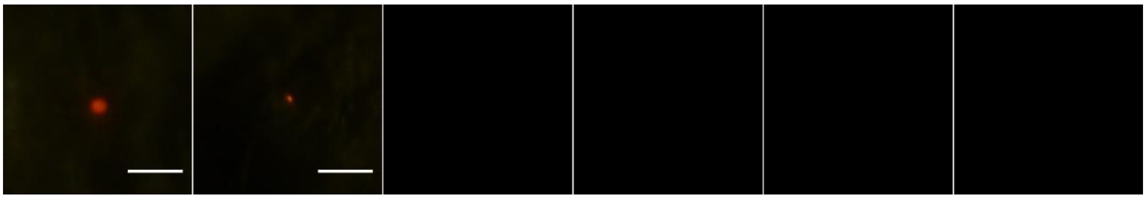

Figure S84. Fluorescent particles (suspected microplastics), with a 50 µm scale, observed in 23% of the filter (without blank subtraction) of A142.

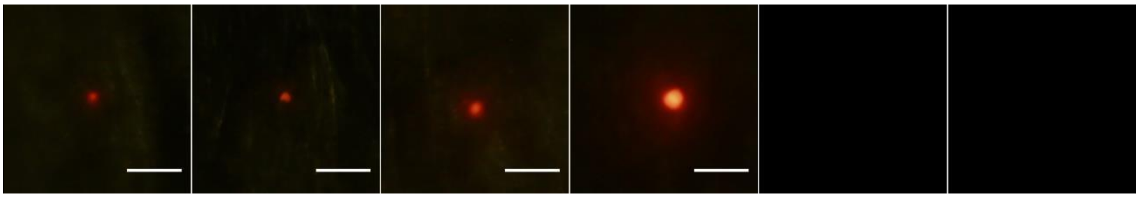

Figure S85. Fluorescent particles (suspected microplastics), with a 50 µm scale, observed in 23% of the filter (without blank subtraction) of A144.

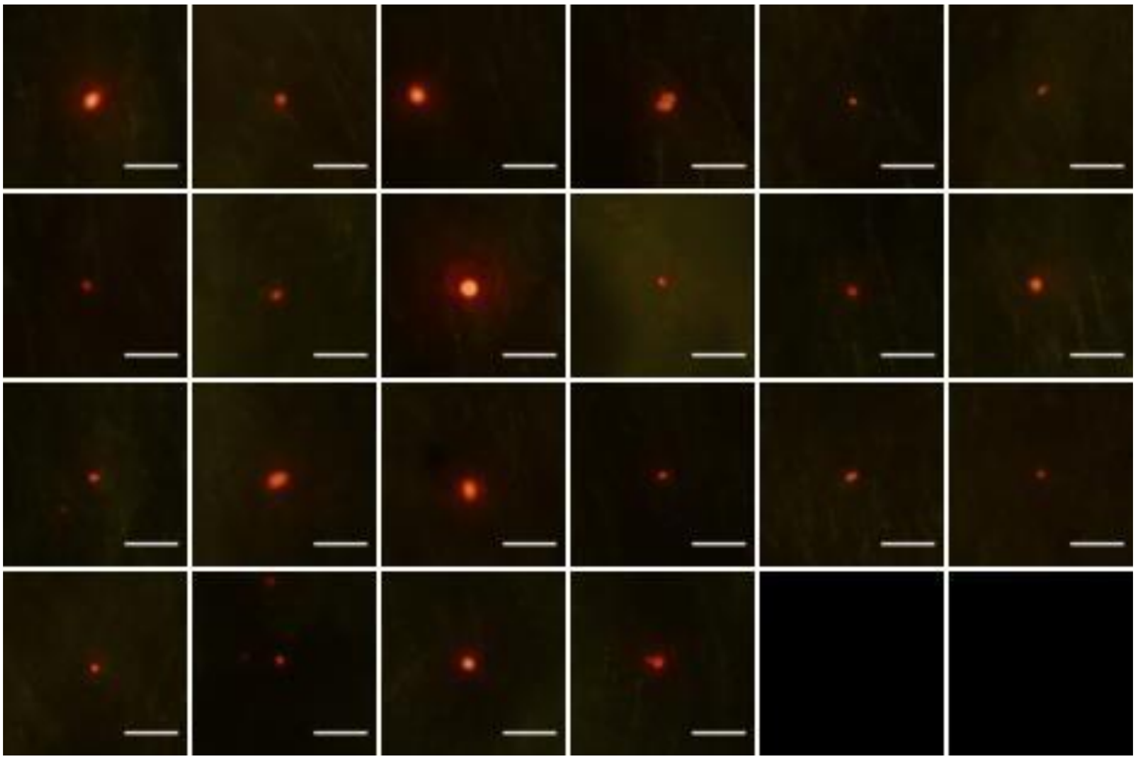

Figure S86. Fluorescent particles (suspected microplastics), with a 50 µm scale, observed in 23% of the filter (without blank subtraction) of A145.

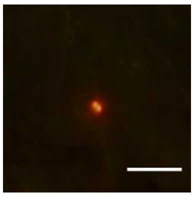

Figure S87. Fluorescent particles (suspected microplastics), with a 50 µm scale, observed in 23% of the filter (without blank subtraction) of A146.

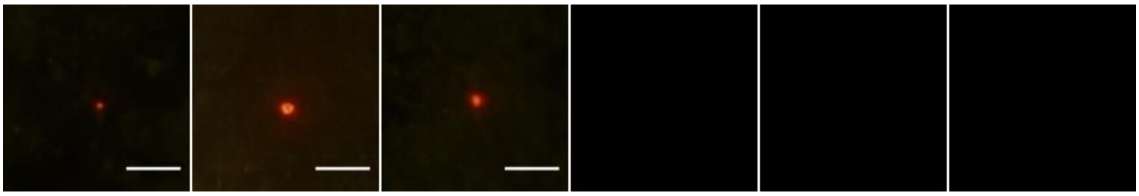

Figure S88. Fluorescent particles (suspected microplastics), with a 50 µm scale, observed in 23% of the filter (without blank subtraction) of A148.

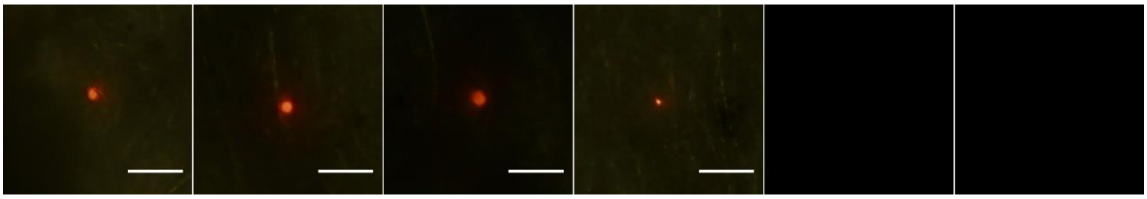

Figure S89. Fluorescent particles (suspected microplastics), with a 50 µm scale, observed in 23% of the filter (without blank subtraction) of A149.

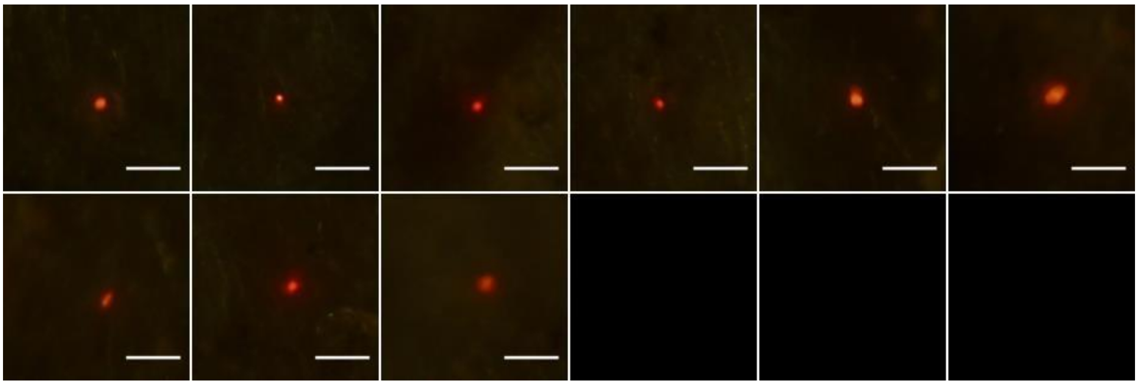

Figure S90. Fluorescent particles (suspected microplastics), with a 50 µm scale, observed in 23% of the filter (without blank subtraction) of A150.

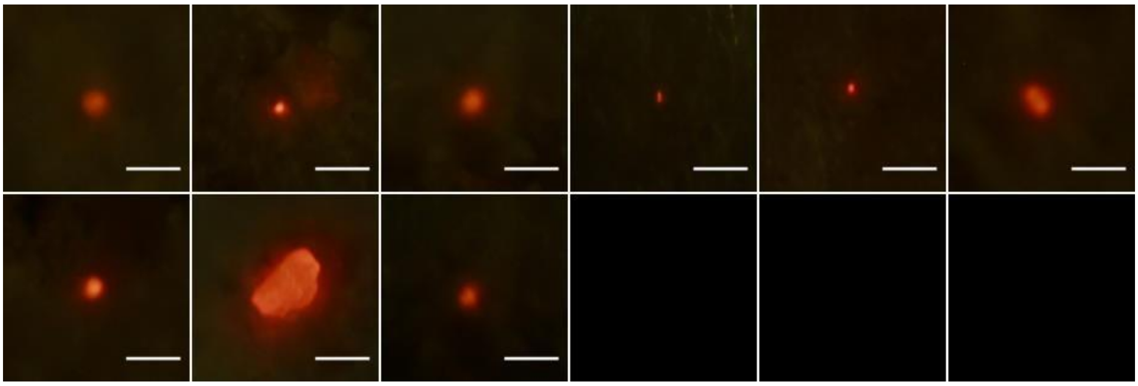

Figure S91. Fluorescent particles (suspected microplastics), with a 50 µm scale, observed in 23% of the filter (without blank subtraction) of A151.

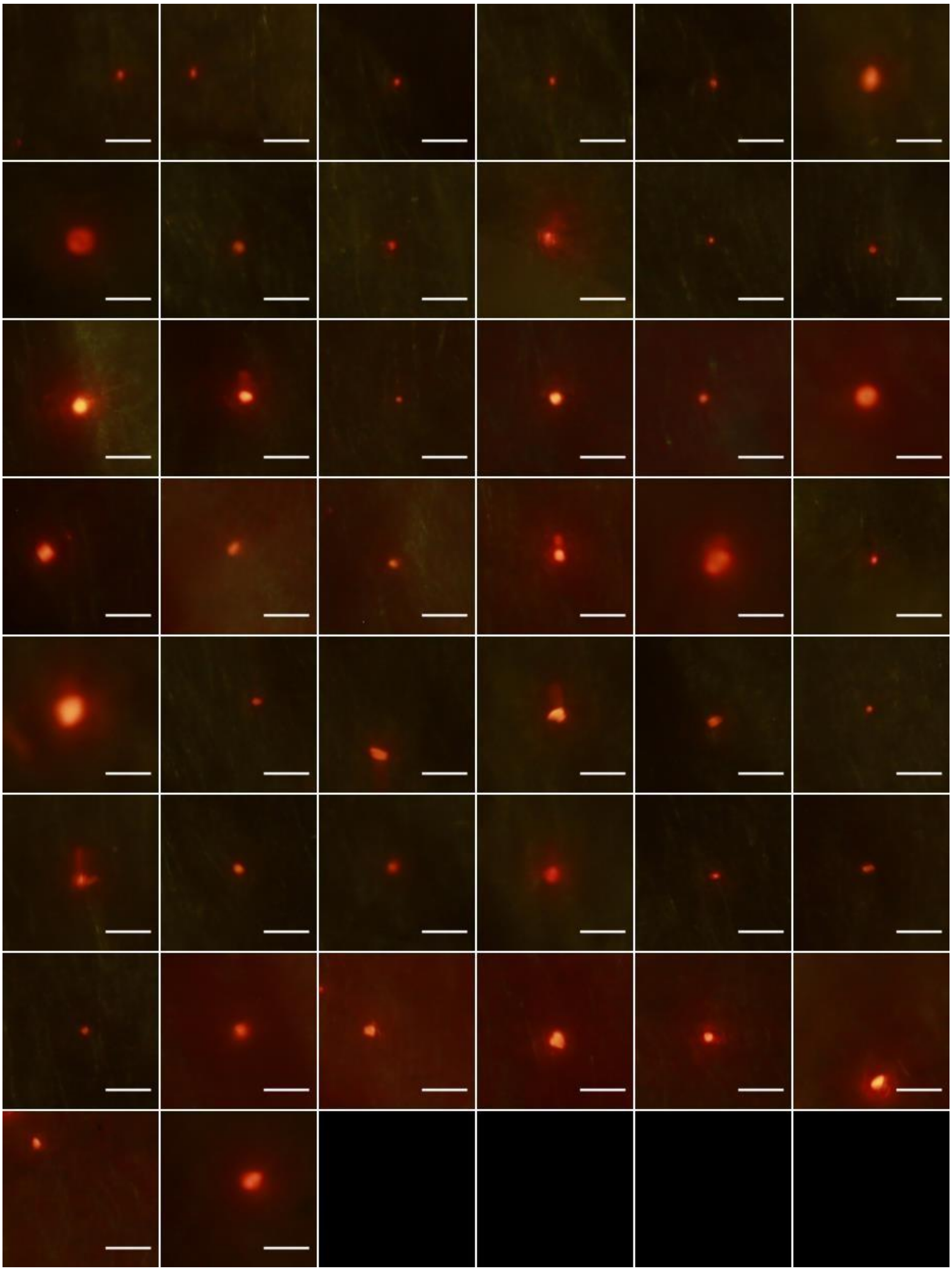

Figure S92. Fluorescent particles (suspected microplastics), with a 50 µm scale, observed in 23% of the filter (without blank subtraction) of A152.

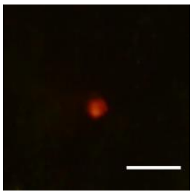

Figure S93. Fluorescent particles (suspected microplastics), with a 50 µm scale, observed in 23% of the filter (without blank subtraction) of A153.

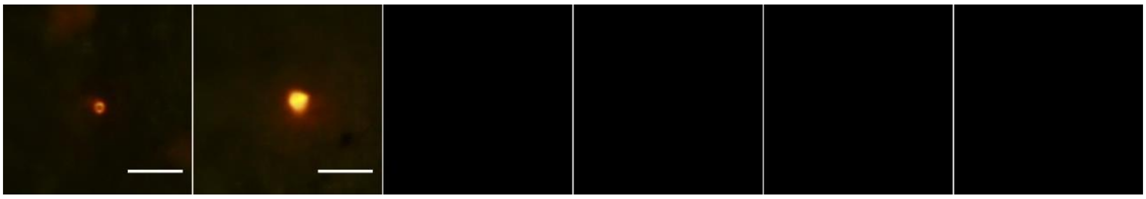

Figure S94. Fluorescent particles (suspected microplastics), with a 50 µm scale, observed in 23% of the filter (without blank subtraction) of A154.

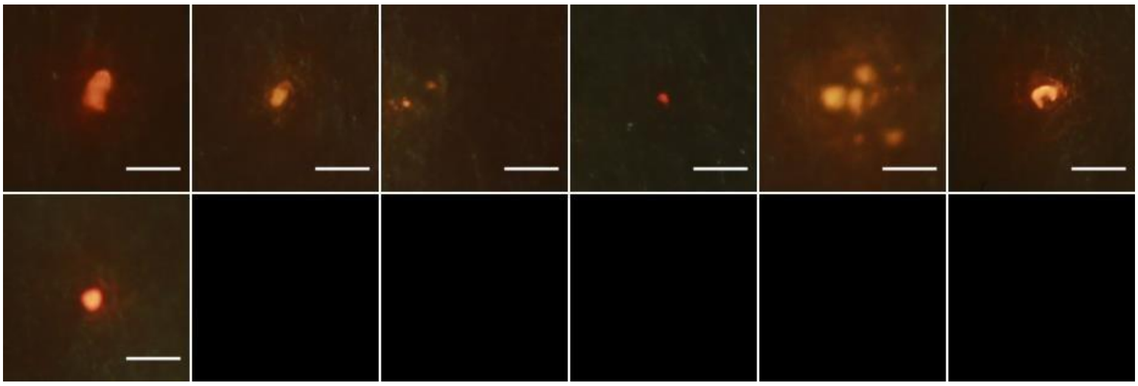

Figure S95. Fluorescent particles (suspected microplastics), with a 50 µm scale, observed in 23% of the filter (without blank subtraction) of A155.

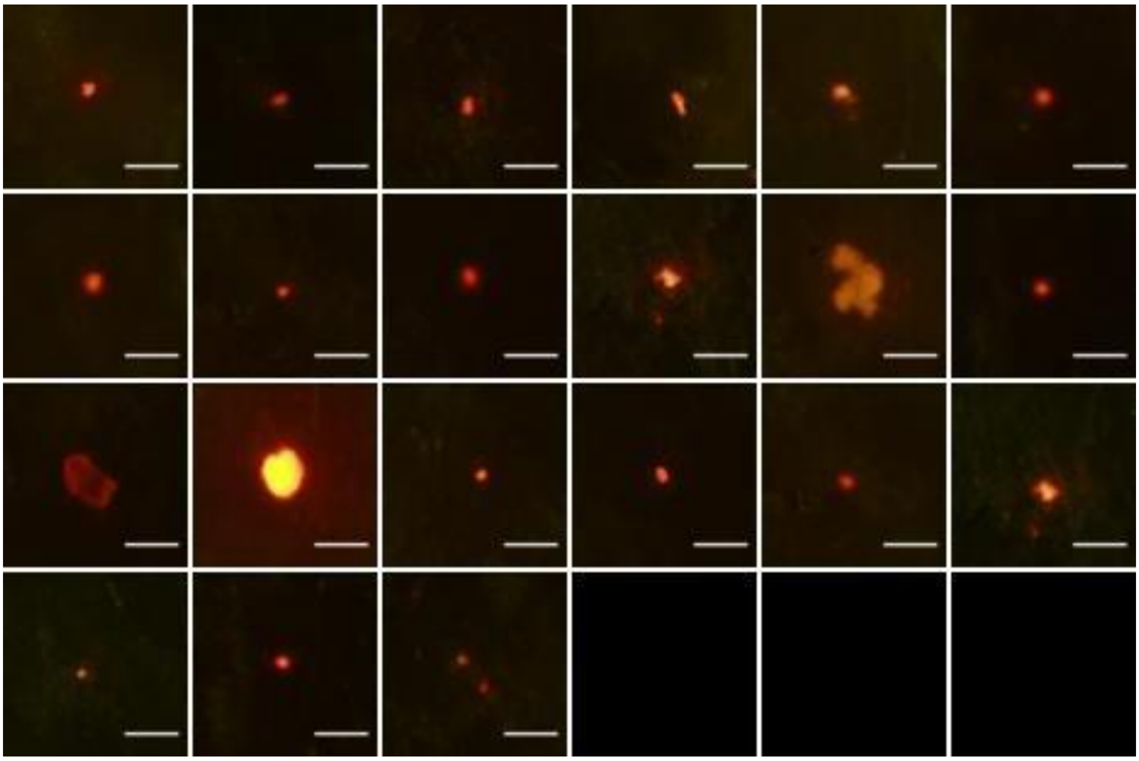

Figure S96. Fluorescent particles (suspected microplastics), with a 50 µm scale, observed in 23% of the filter (without blank subtraction) of A159.

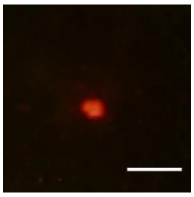

Figure S97. Fluorescent particles (suspected microplastics), with a 50 µm scale, observed in 23% of the filter (without blank subtraction) of A160.

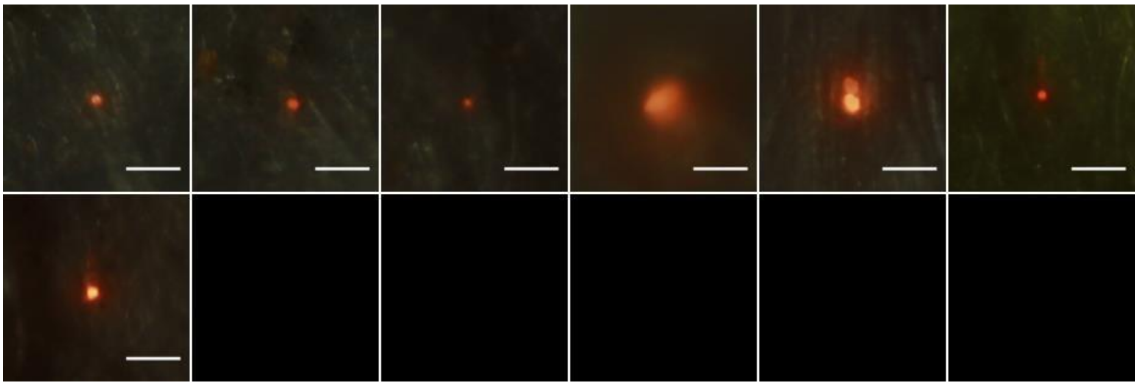

Figure S98. Fluorescent particles (suspected microplastics), with a 50 µm scale, observed in 23% of the filter (without blank subtraction) of A164.

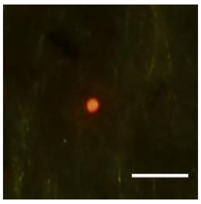

Figure S99. Fluorescent particles (suspected microplastics), with a 50 µm scale, observed in 23% of the filter (without blank subtraction) of A165.

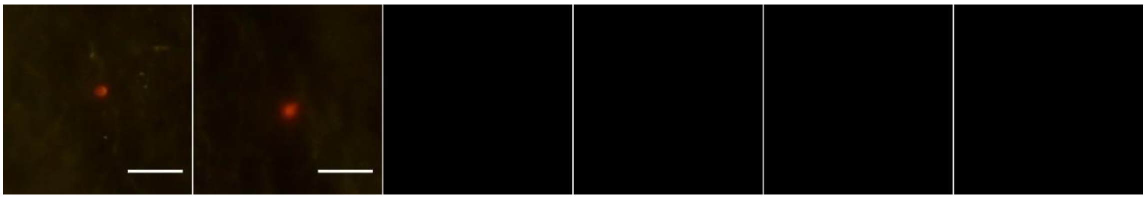

Figure S100. Fluorescent particles (suspected microplastics), with a 50 µm scale, observed in 23% of the filter (without blank subtraction) of A168.

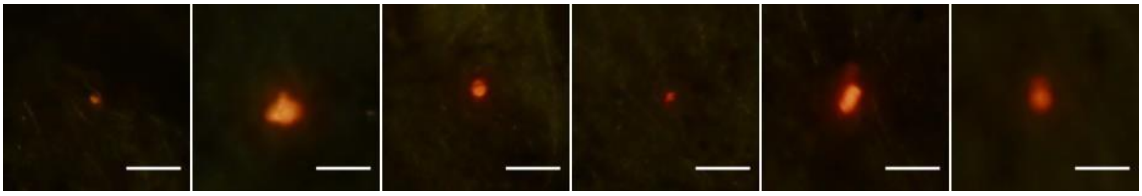

Figure S101. Fluorescent particles (suspected microplastics), with a 50 µm scale, observed in 23% of the filter (without blank subtraction) of A169.

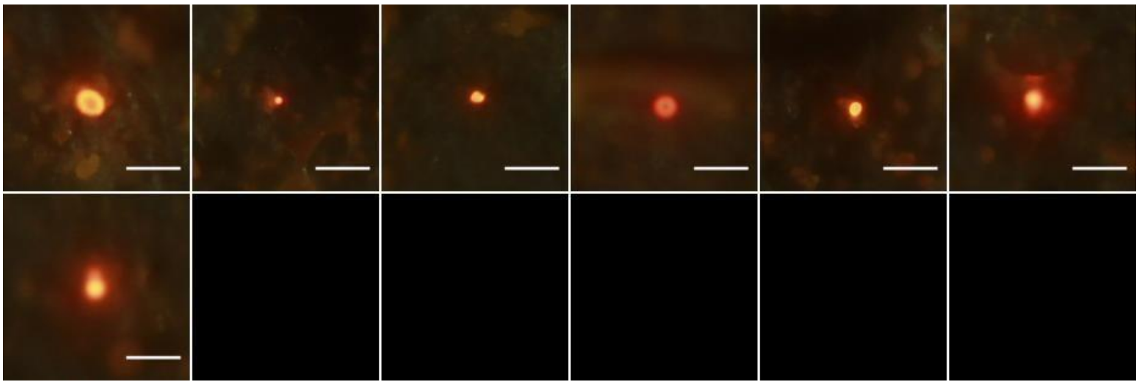

Figure S102. Fluorescent particles (suspected microplastics), with a 50 µm scale, observed in 23% of the filter (without blank subtraction) of A170.

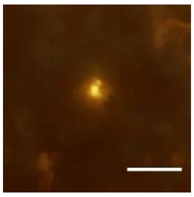

Figure S103. Fluorescent particles (suspected microplastics), with a 50 µm scale, observed in 23% of the filter (without blank subtraction) of A171.

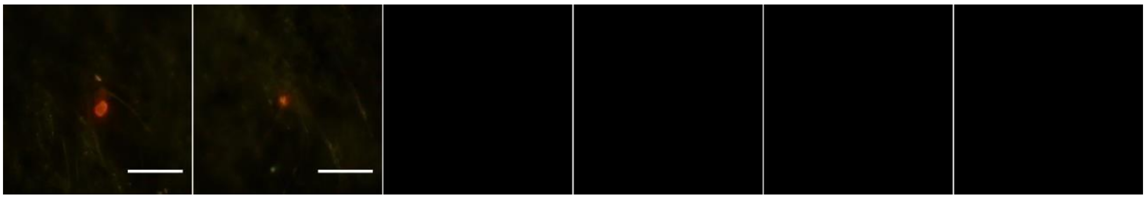

Figure S104. Fluorescent particles (suspected microplastics), with a 50 µm scale, observed in 23% of the filter (without blank subtraction) of A174.

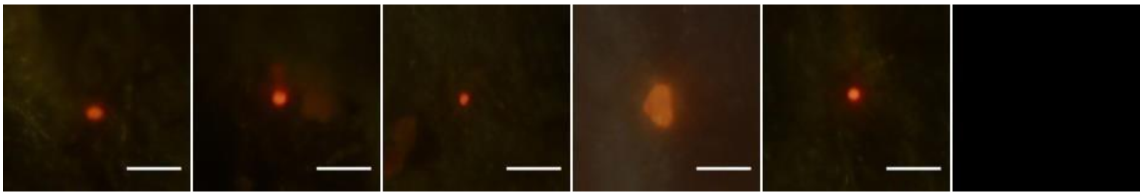

Figure S105. Fluorescent particles (suspected microplastics), with a 50 µm scale, observed in 23% of the filter (without blank subtraction) of A175.

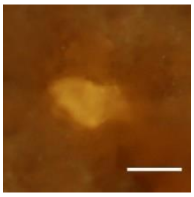

Figure S106. Fluorescent particles (suspected microplastics), with a 50 µm scale, observed in 23% of the filter (without blank subtraction) of A176.

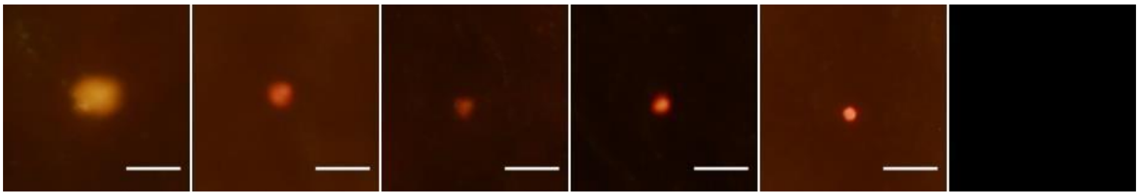

Figure S107. Fluorescent particles (suspected microplastics), with a 50 µm scale, observed in 23% of the filter (without blank subtraction) of A178.

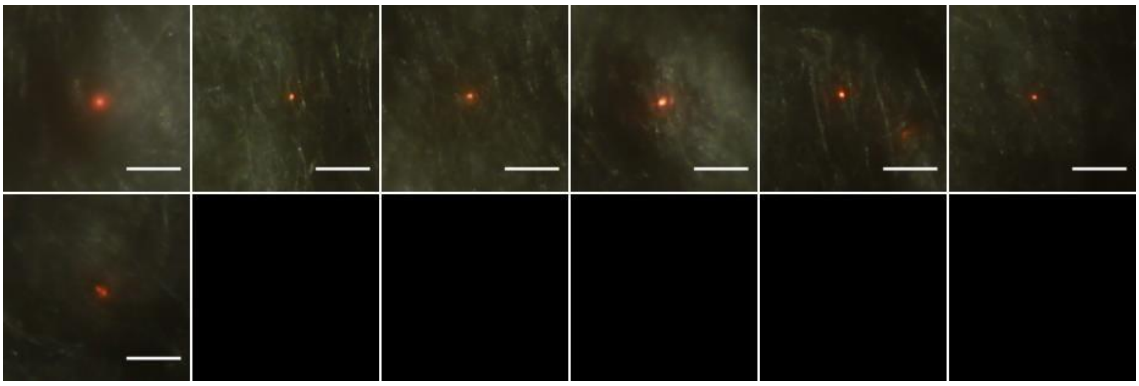

Figure S108. Fluorescent particles (suspected microplastics), with a 50 µm scale, observed in 23% of the filter (without blank subtraction) of A179.

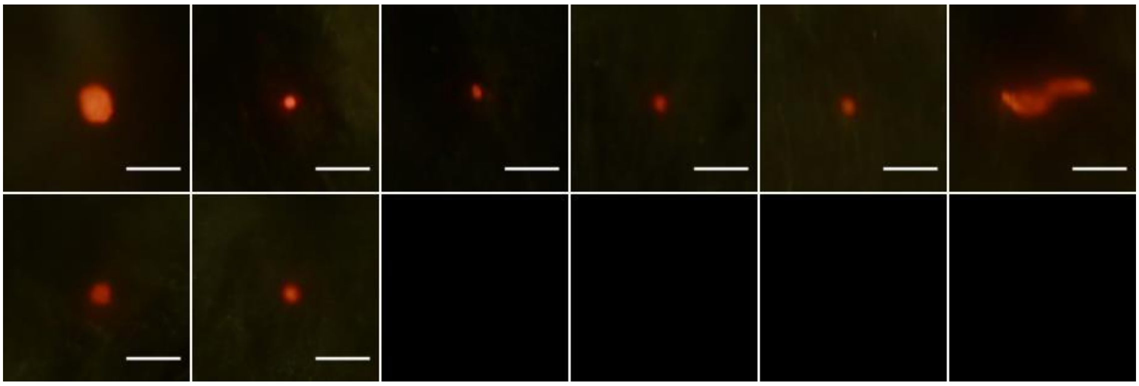

Figure S109. Fluorescent particles (suspected microplastics), with a 50 µm scale, observed in 23% of the filter (without blank subtraction) of A180.

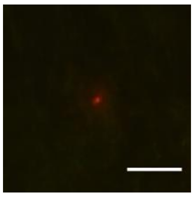

Figure S110. Fluorescent particles (suspected microplastics), with a 50 μm scale, observed in 23% of the filter (without blank subtraction) of A182.

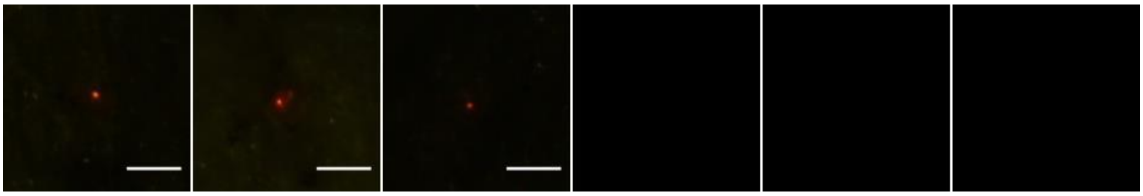

Figure S111. Fluorescent particles (suspected microplastics), with a 50 μm scale, observed in 23% of the filter (without blank subtraction) of A185.

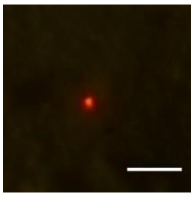

Figure S112. Fluorescent particles (suspected microplastics), with a 50 μm scale, observed in 23% of the filter (without blank subtraction) of A186.

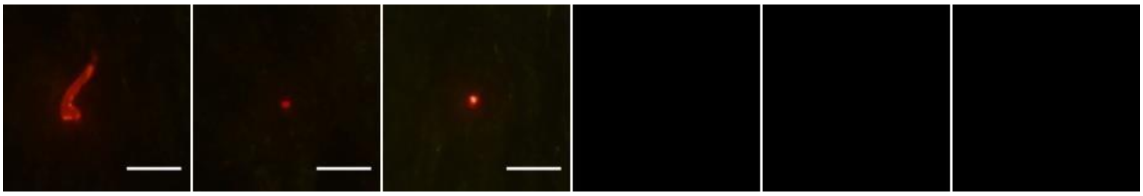

Figure S113. Fluorescent particles (suspected microplastics), with a 50 μm scale, observed in 23% of the filter (without blank subtraction) of A189.

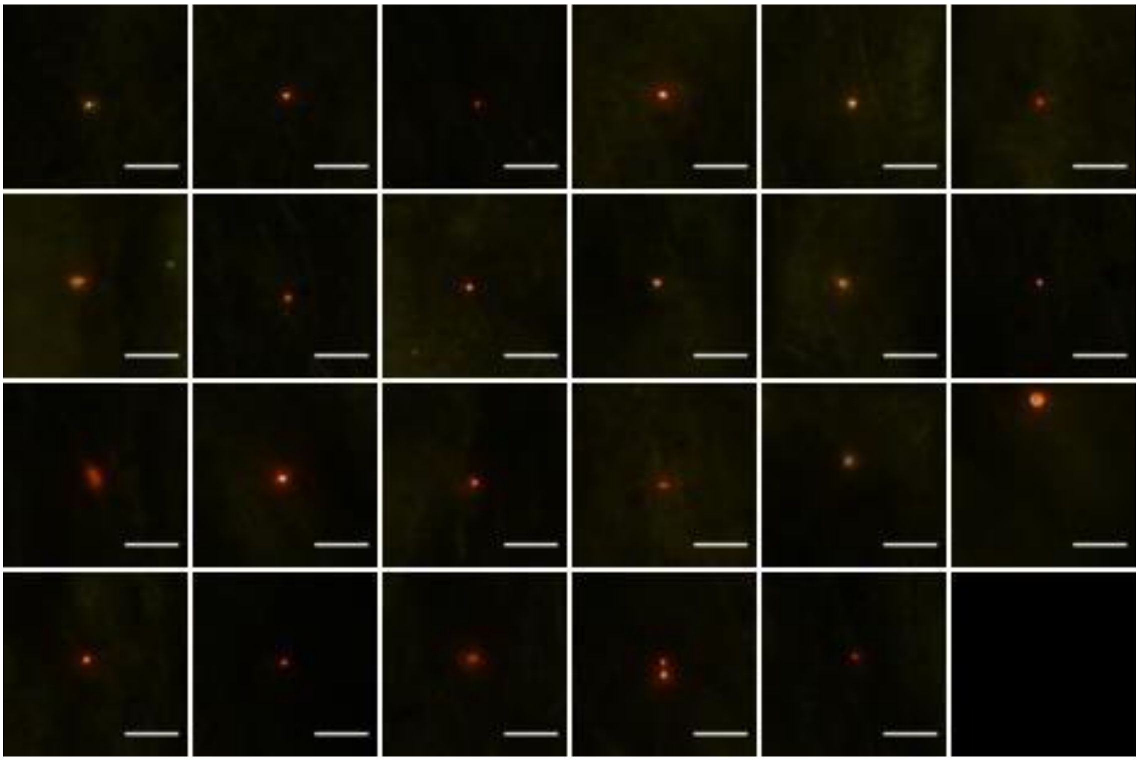

Figure S114. Fluorescent particles (suspected microplastics), with a 50 µm scale, observed in 23% of the filter (without blank subtraction) of A190 (two particles in the fourth panel from the right of the last row).

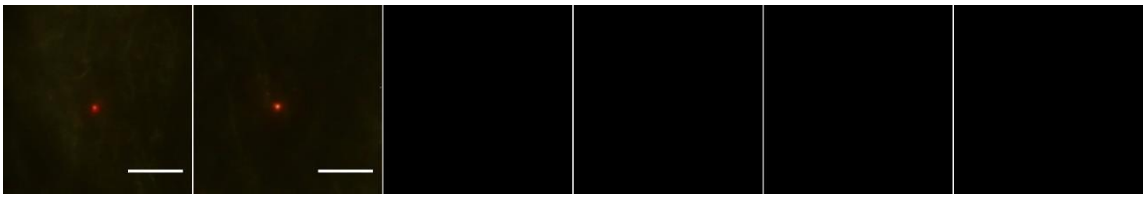

Figure S115. Fluorescent particles (suspected microplastics), with a 50 µm scale, observed in 23% of the filter (without blank subtraction) of A191.

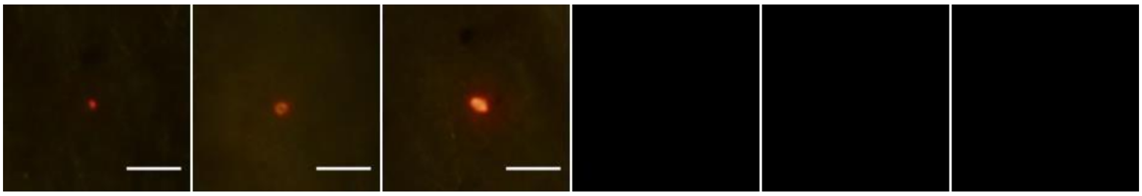

Figure S116. Fluorescent particles (suspected microplastics), with a 50 µm scale, observed in 23% of the filter (without blank subtraction) of A192.

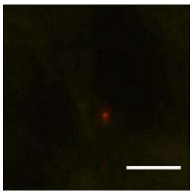

Figure S117. Fluorescent particles (suspected microplastics), with a 50 µm scale, observed in 23% of the filter (without blank subtraction) of A193.

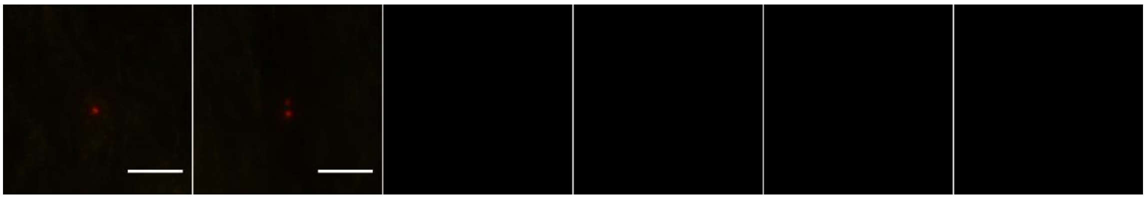

Figure S118. Fluorescent particles (suspected microplastics), with a 50 µm scale, observed in 23% of the filter (without blank subtraction) of A194.

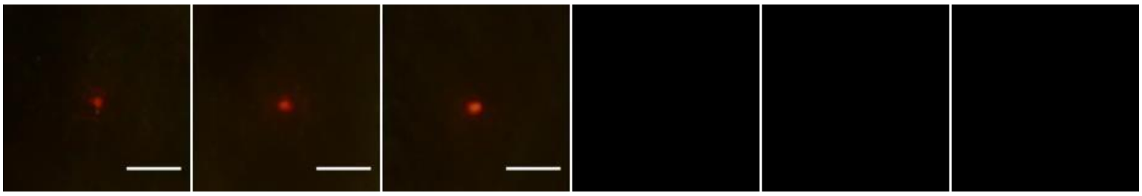

Figure S119. Fluorescent particles (suspected microplastics), with a 50 µm scale, observed in 23% of the filter (without blank subtraction) of A196.

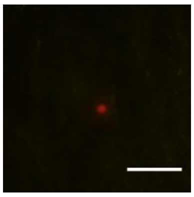

Figure S120. Fluorescent particles (suspected microplastics), with a 50 µm scale, observed in 23% of the filter (without blank subtraction) of A199.

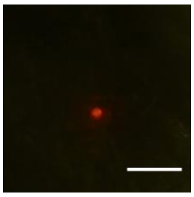

Figure S121. Fluorescent particles (suspected microplastics), with a 50 µm scale, observed in 23% of the filter (without blank subtraction) of A200.

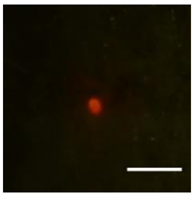

Figure S122. Fluorescent particles (suspected microplastics), with a 50 µm scale, observed in 23% of the filter (without blank subtraction) of A202.

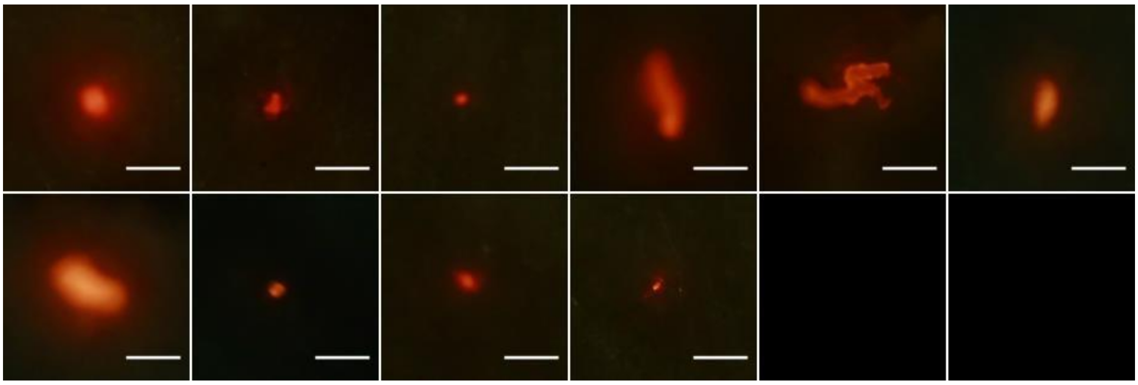

Figure S123. Fluorescent particles (suspected microplastics), with a 50 µm scale, observed in 23% of the filter (without blank subtraction) of A203.

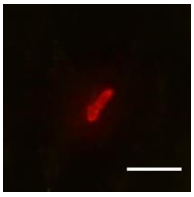

Figure S124. Fluorescent particles (suspected microplastics), with a 50 µm scale, observed in 23% of the filter (without blank subtraction) of A204.

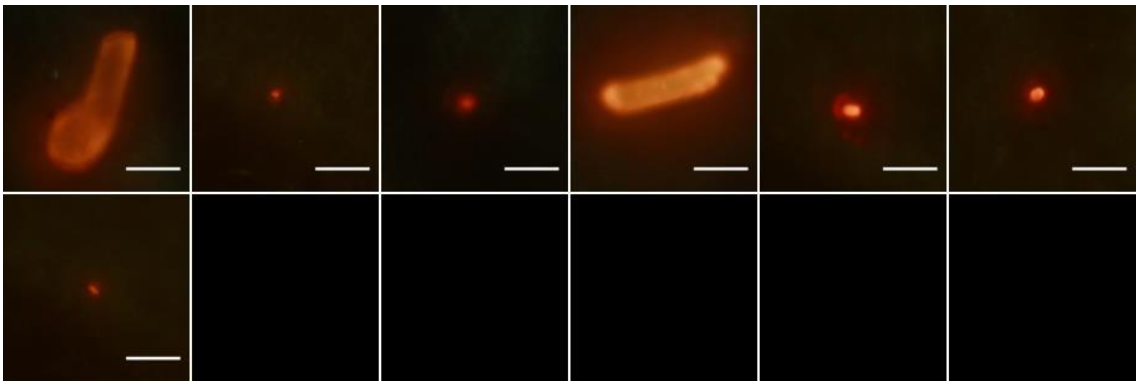

Figure S125. Fluorescent particles (suspected microplastics), with a 50 µm scale, observed in 23% of the filter (without blank subtraction) of A205.

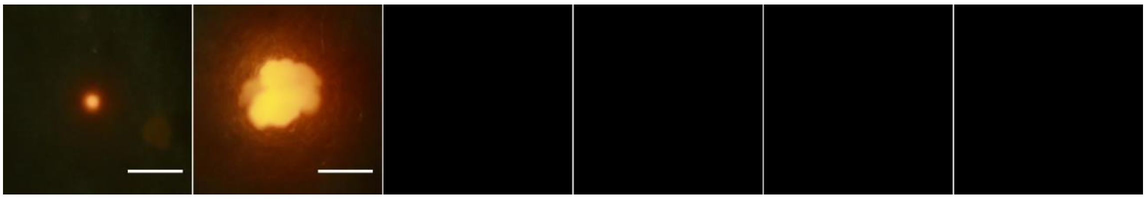

Figure S126. Fluorescent particles (suspected microplastics), with a 50 µm scale, observed in 23% of the filter (without blank subtraction) of A206.

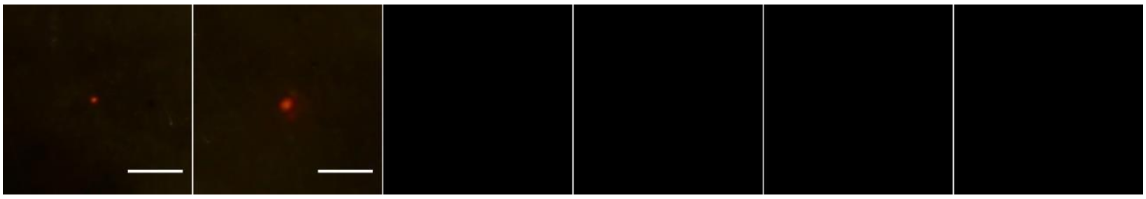

Figure S127. Fluorescent particles (suspected microplastics), with a 50 µm scale, observed in 23% of the filter (without blank subtraction) of A207.

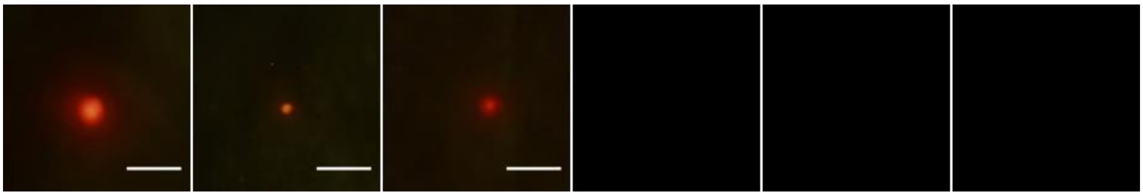

Figure S128. Fluorescent particles (suspected microplastics), with a 50 µm scale, observed in 23% of the filter (without blank subtraction) of A208.

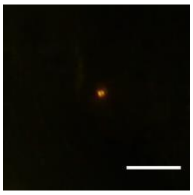

Figure S129. Fluorescent particles (suspected microplastics), with a 50 µm scale, observed in 23% of the filter (without blank subtraction) of A210.

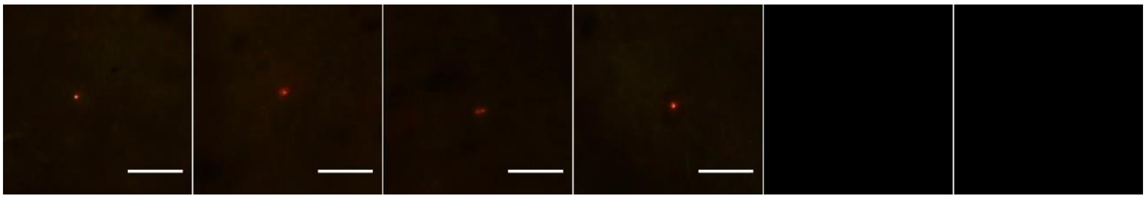

Figure S130. Fluorescent particles (suspected microplastics), with a 50 µm scale, observed in 23% of the filter (without blank subtraction) of A212.

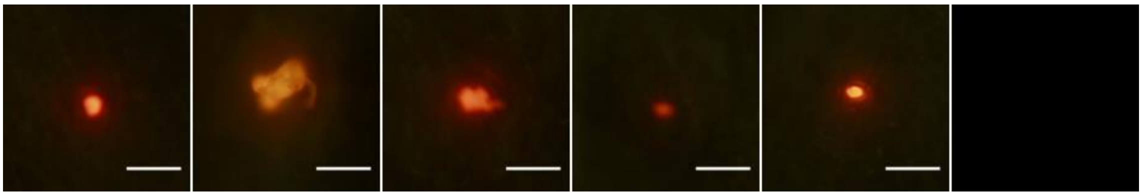

Figure S131. Fluorescent particles (suspected microplastics), with a 50 µm scale, observed in 23% of the filter (without blank subtraction) of A214.

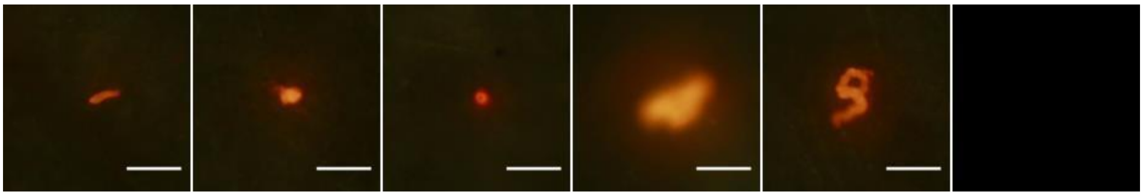

Figure S132. Fluorescent particles (suspected microplastics), with a 50 µm scale, observed in 23% of the filter (without blank subtraction) of A215.

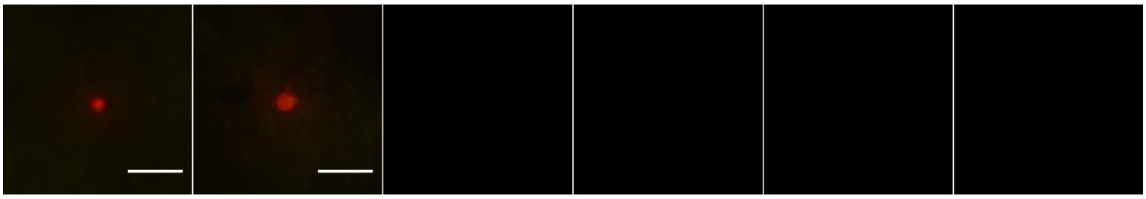

Figure S133. Fluorescent particles (suspected microplastics), with a 50 µm scale, observed in 23% of the filter (without blank subtraction) of A216.

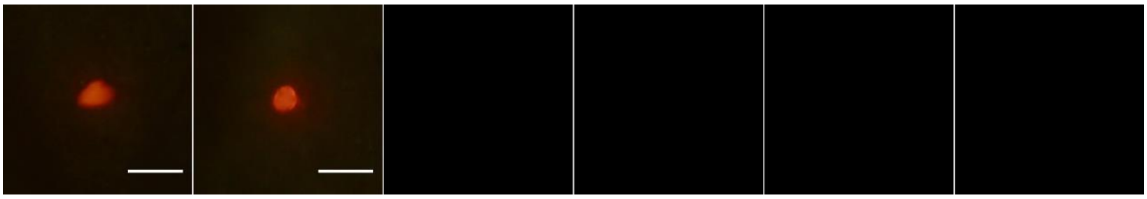

Figure S134. Fluorescent particles (suspected microplastics), with a 50 µm scale, observed in 23% of the filter (without blank subtraction) of A217.

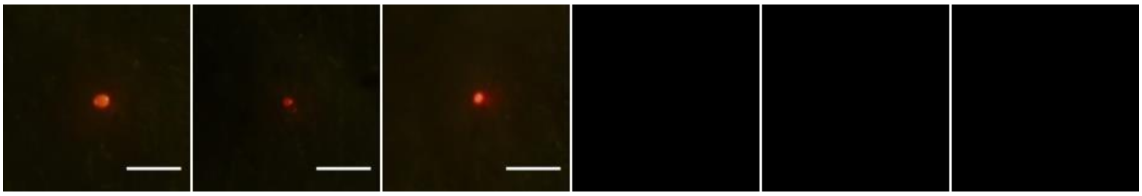

Figure S135. Fluorescent particles (suspected microplastics), with a 50 µm scale, observed in 23% of the filter (without blank subtraction) of A218.

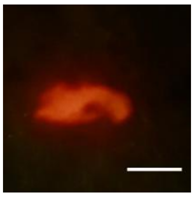

Figure S136. Fluorescent particles (suspected microplastics), with a 50 µm scale, observed in 23% of the filter (without blank subtraction) of A220.

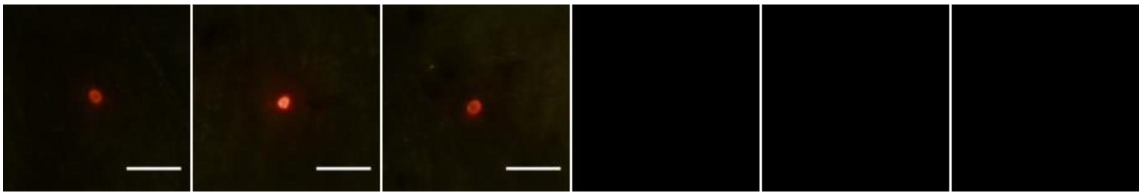

Figure S137. Fluorescent particles (suspected microplastics), with a 50 µm scale, observed in 23% of the filter (without blank subtraction) of A223.

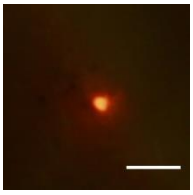

Figure S138. Fluorescent particles (suspected microplastics), with a 50 µm scale, observed in 23% of the filter (without blank subtraction) of A227.

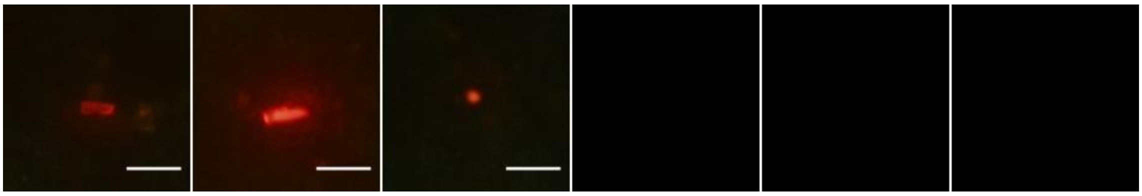

Figure S139. Fluorescent particles (suspected microplastics), with a 50 µm scale, observed in 23% of the filter (without blank subtraction) of A230.

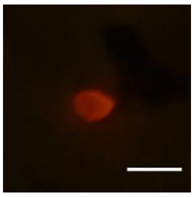

Figure S140. Fluorescent particles (suspected microplastics), with a 50 µm scale, observed in 23% of the filter (without blank subtraction) of A231.

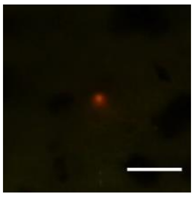

Figure S141. Fluorescent particles (suspected microplastics), with a 50 µm scale, observed in 23% of the filter (without blank subtraction) of A232.

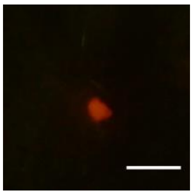

Figure S142. Fluorescent particles (suspected microplastics), with a 50 µm scale, observed in 23% of the filter (without blank subtraction) of A235.

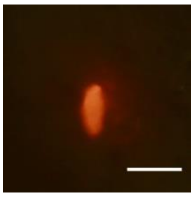

Figure S143. Fluorescent particles (suspected microplastics), with a 50 µm scale, observed in 23% of the filter (without blank subtraction) of A236.

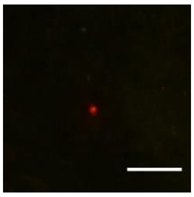

Figure S144. Fluorescent particles (suspected microplastics), with a 50 µm scale, observed in 23% of the filter (without blank subtraction) of A239.

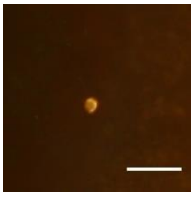

Figure S145. Fluorescent particles (suspected microplastics), with a 50 µm scale, observed in 23% of the filter (without blank subtraction) of A241.

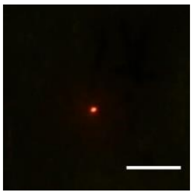

Figure S146. Fluorescent particles (suspected microplastics), with a 50 µm scale, observed in 23% of the filter (without blank subtraction) of A247.

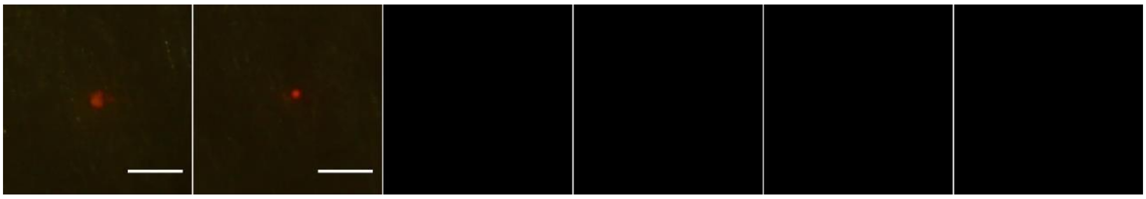

Figure S147. Fluorescent particles (suspected microplastics), with a 50 µm scale, observed in 23% of the filter of blank 1 (batch 2).

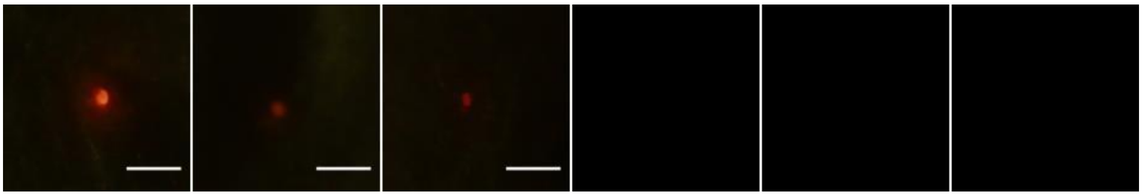

Figure S148. Fluorescent particles (suspected microplastics), with a 50 µm scale, observed in 23% of the filter of blank 4 (batch 2).

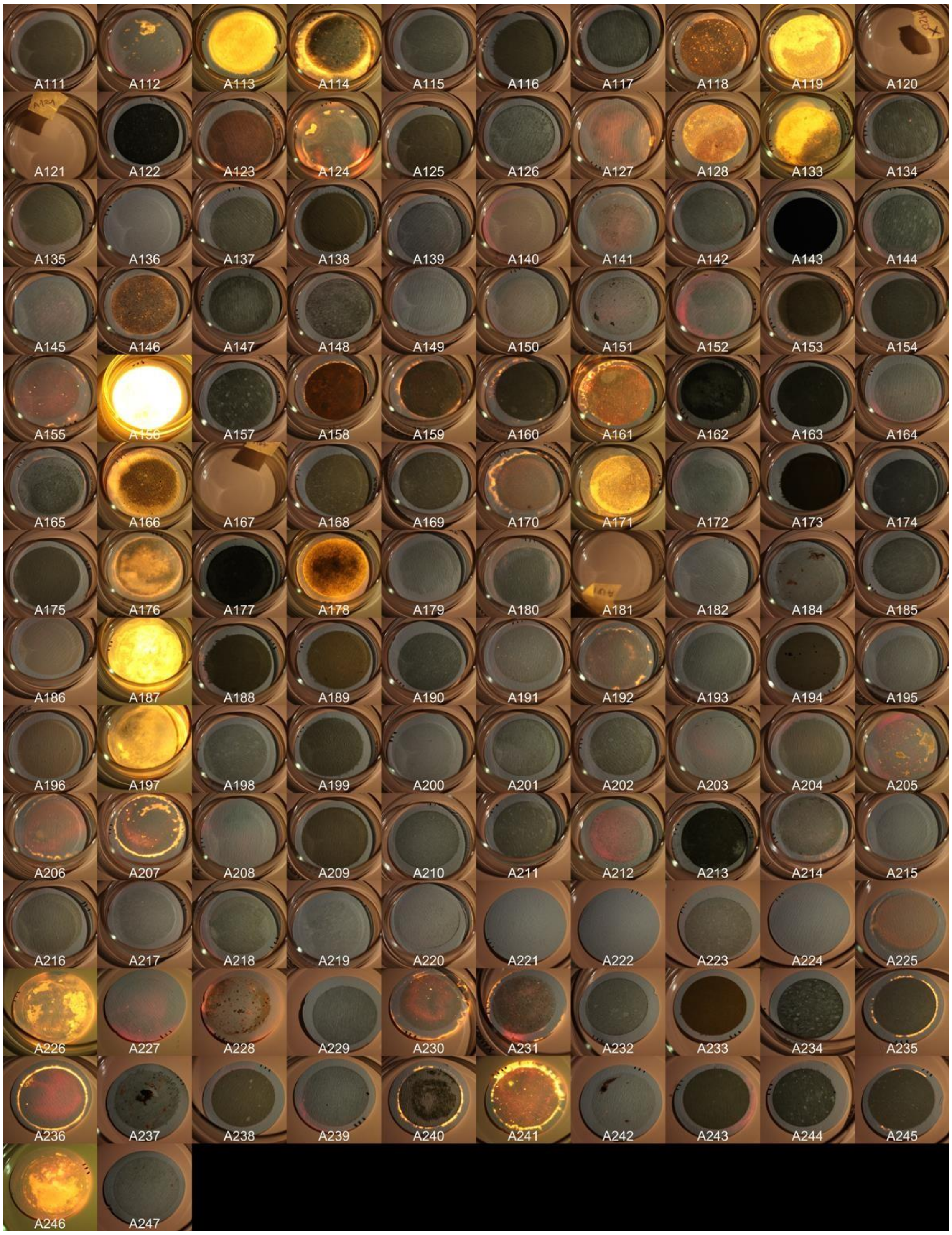

Figure S149. Filter membranes of the batch 2 of companion animal samples.

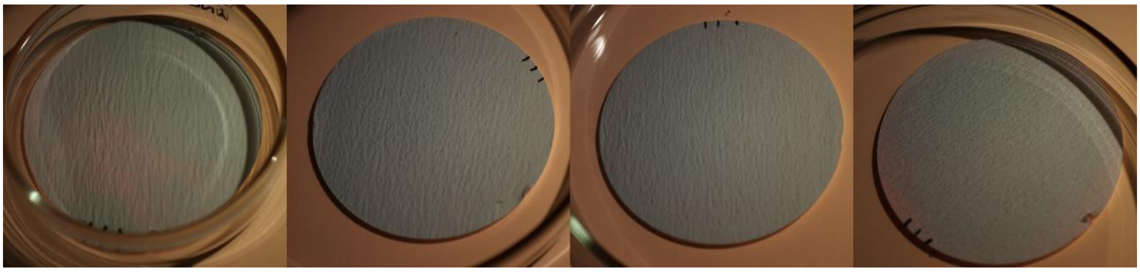

Figure S150. Filter membranes of batch 2 blanks.
